# A Prebiotic Synthesis of Canonical Pyrimidine and Purine Ribonucleotides

**DOI:** 10.1089/ast.2018.1935

**Published:** 2019-04-25

**Authors:** Hyo-Joong Kim, Justin Kim

**Affiliations:** ^1^ Firebird Biomolecular Sciences LLC, Alachua, Florida.; ^2^ Buchholz High School, Gainesville, Florida.

**Keywords:** Prebiotic chemistry, Glycosidic bond formation, Ribose cyclic phosphate

## Abstract

The “RNA first” model for the origin of life holds that RNA emerged spontaneously on early Earth and developed into life through its dual capabilities for genetics and catalysis. The model's central weakness is the difficulty of making its building blocks, in particular, the glycosidic bond joining nucleobases to ribose. Thus, the focus of much of the modern literature on the topic is directed toward solving this difficulty and includes elegant, though indirect, methods for making this bond. Here, we report that the glycosidic bond in canonical pyrimidine and purine ribonucleotides can be formed by direct coupling of cyclic carbohydrate phosphates with free nucleobases, all reported to be available by experimentally supported pathways that might have operated on early Earth.

## 1. Introduction

Because of its dual capabilities as a genetic molecule and as a catalytic molecule (Kruger *et al.,* 1982; Guerrier-Takada *et al.,* 1983), RNA seems to be an ideal first chemical to support Darwinian evolution as life emerged on early Earth. This “RNA first” hypothesis (Gilbert, 1986) requires, of course, that RNA was synthesized spontaneously on early Earth by abiotic pathways. Accordingly, many groups, using “RNA first” as their working hypothesis, have attempted to combine laboratory chemistry with models for the early geology of Earth and its early atmospheric evolution to yield building blocks for RNA (Orgel, 2004; Harrison *et al.,* 2005; Ruiz-Mirazo *et al.,* 2014).

Models to make various components of the building blocks of RNA have developed over the years. Routes to make the canonical nucleobases have been known since the 1960s, including adenine (Oro, 1961), guanine (Levy *et al.,* 1999), cytosine, and uracil (Robertson and Miller, 1995). Likewise, prebiotic routes to ribose involving borate minerals are proposed (Ricardo *et al.,* 2004; Kim *et al.,* 2011; Furukawa and Kakegawa, 2017). Also, the prebiotic synthesis of enantiomeric enriched carbohydrates (Breslow *et al.,* 2013) and 2-deoxy ribose are reported (Steer *et al.,* 2017).

However, prebiotic routes to nucleosides, which connect the ribose to the nucleobases, have been a prominent missing link in the abiotic synthesis of RNA. Direct glycosidic bond formation was first studied by the Orgel group, which showed that the incubation of ribose with purine nucleobases in the presence of Mg^2+^ in dry state yielded purine nucleosides, adenosine, and guanosine (Fuller *et al.,* 1972). However, the direct condensation of ribose and these purines also gave unwanted isomers as major products and failed entirely for the pyrimidines, cytosine, and uracil.

This drove Sutherland, Carell, and others in the field to consider indirect approaches. For example, Carell's group reported the direct condensation of formylated aminopyrimidines with ribose to yield a product that, although not a canonical nucleoside, could be further elaborated to yield adenosine and guanosine, without phosphates but here with regiochemical control (Becker *et al.,* 2016). Sutherland and Powner focused on the pyrimidine nucleotides (as 2′,3′-phosphates), forming a precursor of these from fragments of both the nucleobase and the ribose. The product was then elaborated to complete both the sugar and the nucleobase, together with photochemical conversion of the unwanted stereoisomers (Powner *et al.,* 2009; Stairs *et al.,* 2017; Xu *et al.,* 2017). Also, photoanomerization of α-cytidine 2′-phosphate provided a mixture of β-cytidine and uridine 2′-phosphate (Powner and Sutherland, 2008).

Direct syntheses have been reported recently by Nam *et al.* (2017) and Nam *et al.* (2018), who sought to phosphorylate ribose followed by further reaction with nucleobases to form nucleosides in microdroplets in acidic condition. Regiochemistry as well as stereochemistry of the nucleosides was not known due to the limitation of their analytical methods, unfortunately. Separately, Saladino *et al.* (2015) reported that various organics, including nucleosides, are made from formamide by high-energy proton irradiation in the presence of meteorites. High-energy proton fluxes are expected, of course, only in outer space (in meteors or comets). Examination of a small set of carbonaceous chondrites finds none of these materials in easily detectable amounts for delivery to Earth, although ribose and other carbohydrates are made in cosmogenic ice simulants (Meinert *et al.,* 2016).

## 2. Materials and Methods

### 2.1. Materials

Ribose 1-phosphate was obtained from Toronto Research Chemicals. Nucleoside starting materials for the chemical synthesis of ribonucleoside 2′-phosphate were obtained from Carbosynth. All other chemicals were obtained from Sigma-Aldrich and TCI and used as received.

### 2.2. Methods

The reaction of ribose 1,2-cyclic phosphate **3** and nucleobases was conducted in an Eppendorf tube containing ribose 1,2-cyclic phosphate **3** (5 μL, 15 mM), nucleobases (20 μL, 3.75 mM in case of 1:1 molar ratio), and calcium chloride (8 μL,15 mM) in aqueous solution. The pH of the solution was 6.5. The mixture was heated with lids open until all the water evaporated. The residues were further heated at 100–125°C for 3–96 h. It was redissolved in water (0.3 mL) and analyzed by ion exchange HPLC.

Ion exchange high-performance liquid chromatography (HPLC) analysis was done with a DNAPac PA-100 column (4 × 250 mm; Thermo Scientific) on a Waters 2695 separation module equipped with a 996 photodiode array detector. The column was eluted with a gradient of (A) water and (B) 1 M of ammonium bicarbonate. The elution program created a linear gradient started from 100% (by volume) A to 80% A at 15 min with a flow rate of 0.5 mL/min. Peak detection and integration were conducted with the signal at 260 nm. Full UV spectra (210–400 nm) were also obtained. Reverse-phase HPLC analysis was done with a C-18 reversed-phase narrow bore column (3 mm i.d., 150 mm length, 5 μm; SunFire; Waters) on a Waters 2695 separation module equipped with a 996 photodiode array detector. The column was eluted with a gradient of (A) aqueous 25 mM triethylammonium acetate and (B) 100% acetonitrile. The elution program created a linear gradient started from 100% (by volume) A to 85% A at 10 min with a flow rate of 0.5 mL/min. Peak detection and integration were conducted with the signal at 260 nm. Full UV spectra (210–350 nm) were also obtained. Preparative HPLC purification was achieved with an ion exchange column (22 mm i.d., 250 mm length, 5 μm; DNAPac PA-100; Thermo Fisher Scientific) on a Waters Delta 600 module. The column was eluted with a gradient of (A) water and (B) 1 M of ammonium bicarbonate. The elution program created a linear gradient started from 100% A to 80% A : 20% B at 15 min with a flow rate of 10 mL/min. Peak detection was conducted by using the 260 nm absorbance. Nuclear magnetic resonance (NMR) spectra were recorded in deuterium oxide on a Varian Mercury 300 NMR spectrometer. High-resolution mass spectrometry was conducted on Agilent 6220 Time-of-Flight connected with an Agilent 1100 series system that consisted of a G13793 degasser and a G1312B binary pump with electrospray ionization in negative mode.

Ribose 1,2-cyclic phosphate **3** was prepared following the literature method (Fathi and Jordan, 1986). Specifically, dicyclohexylcarbodiimide (40 mg) in *tert*-butyl alcohol (1.9 mL) was added to a stirred solution of D-ribofuranosyl-*l*-phosphate biscyclohexylammonium salt (20 mg) in formamide (0.6 mL), followed by 2 N ammonium hydroxide (0.6 mL). The suspension was heated under reflux for 10 h. The *tert*-butyl alcohol was removed at a rotary evaporator. The residual solution was mixed with an equal amount of water and extracted with ether (2 × 5 mL). The aqueous solution was concentrated under a vacuum (0.02 torr) to remove the formamide. The residue was dissolved in water (3 mL, the final concentration of ribose 1,2-cyclic phosphate is 15 mM). Detailed synthetic procedures for the synthesis of ribonucleoside 2′-phosphates, HPLC analysis data, and NMR and mass spectra are available at https://www.liebertpub.com/suppl/doi/10.1089/ast.2018.1935.

## 3. Results

We previously reported prebiotic synthesis of adenosine 2′-phosphate (Kim and Benner, 2017), nicotinamide nucleoside 2′-phosphate (Kim and Benner, 2018), and some noncanonical purine and pyrimidine nucleoside 2′-phosphates (yields range from 15% to 35%) (Kim and Benner, 2017) by the coupling of ribose 1,2-cyclic phosphate **3** and the corresponding nucleobases. Ribose 1,2-cyclic phosphate **3** is available from ribose **1** and amidotriphosphate **2** in 29% along with two other isomers in aqueous solution (Krishnamurthy *et al.,* 2000). Amidotriphosphate **2** is formed by the ammonolysis of cyclic trimetaphosphate, which is easily formed from inorganic phosphate under dehydrating conditions (Pasek *et al.,* 2008). Further, these syntheses showed stereoselectivity, giving only the β-nucleotide due to the block of α-face by the cyclic phosphate (Vorbrüggen and Ruh-Pohlenz, 2000) and the desired N^9^-nucleoside as a major product with adenine. Importantly, the resulting nucleoside 2′-phosphate was transformed to nucleoside 5′-phosphate or nucleoside by the incubation of nucleoside 2′-phosphate with urea and borate (Kim *et al.,* 2016; Kim and Benner, 2017).

**FIG. 1.**
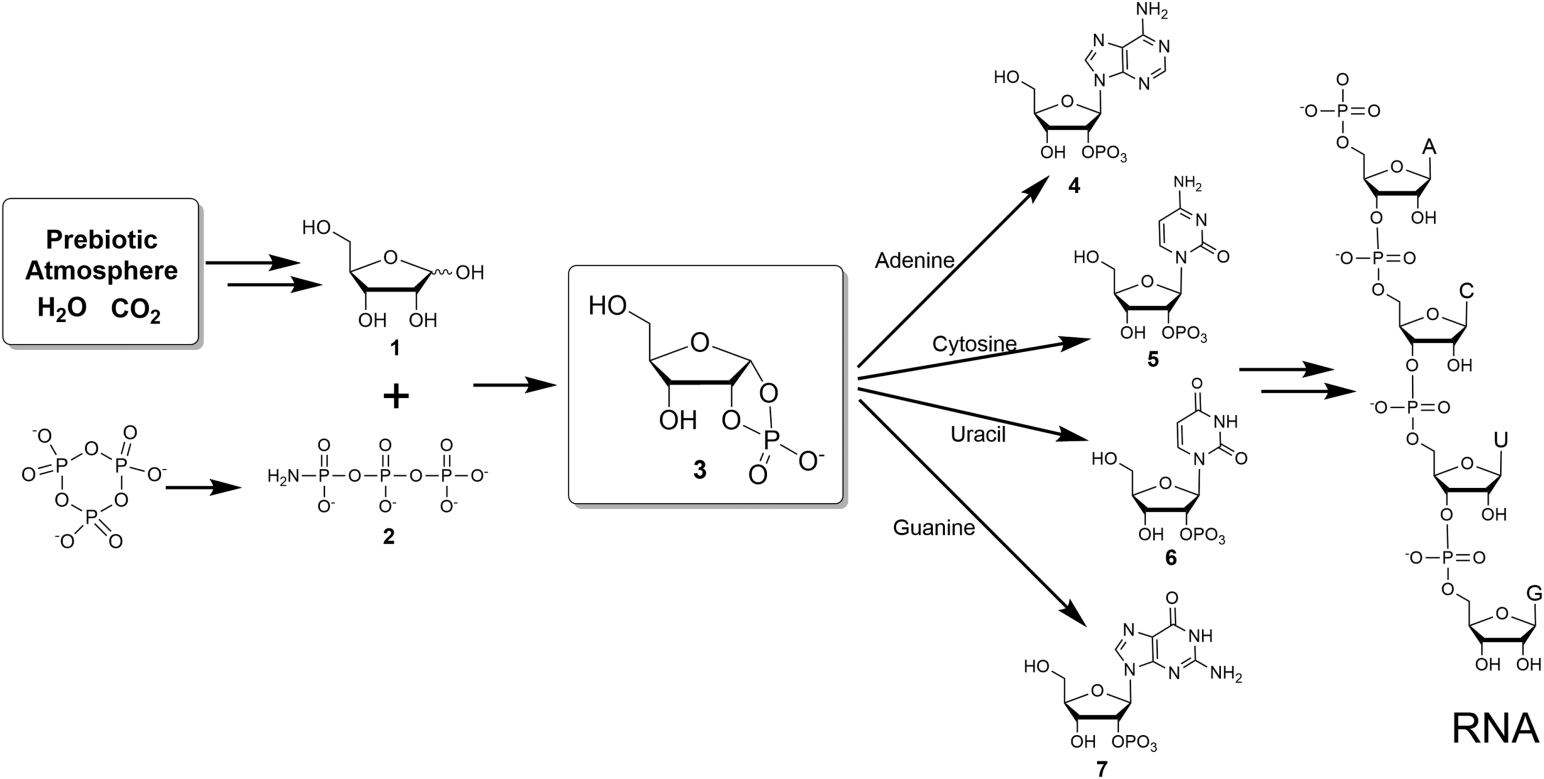
Plausible prebiotic synthetic pathway to RNA. The coupling of ribose 1,2-cyclic phosphate **3** with nucleobases yielded ribonucleoside 2′-phosphates **4**–**7**. Ribose 1,2-cyclic phosphate **3** can be available from ribose **1** and amidotriphosphate **2** in plausible prebiotic conditions.

When combined, these chemistries provide a scenario for a prebiotic source for adenylic acid. We therefore asked whether some combination of these chemistries might yield direct synthesis of all four of the canonical nucleotides. We show that they can be synthesized in plausible prebiotic environments (Fig. 1).

First, coupling of ribose-1,2-cyclic phosphate **3** with cytosine was attempted in the presence of Mg^2+^ in an aqueous mixture evaporated at 80–90°C, the conditions that successfully formed adenosine (Kim and Benner, 2017). Here, however, coupling failed, yielding mostly unreacted cytosine and very small amounts of products that could not be identified initially by HPLC/UV. This was attributed to the lower reactivity of pyrimidine nucleophiles compared to purine nucleophiles, a difference known since the original work of Orgel in the early 1970s (Fuller *et al.,* 1972). An addition of sodium hydroxide did not provide the coupling product with the ribose 1,2-cyclic phosphate **3**, though it did work to generate the analogous threofuranosyluracil nucleotide analog with threose 1,2-cyclic phosphate and uracil (Kim and Benner, 2017).

**FIG. 2.**
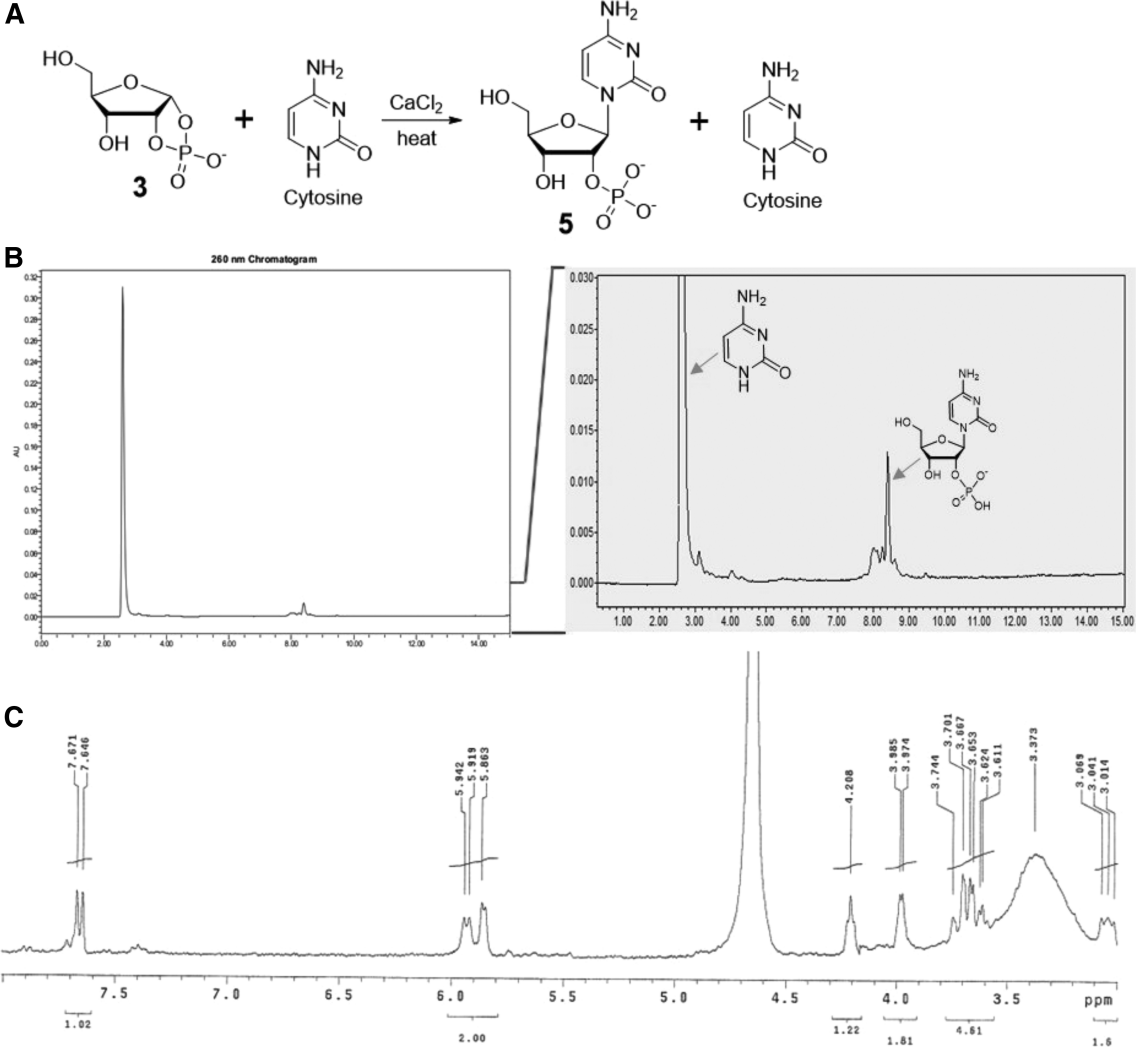
The coupling of ribose 1,2-cyclic phosphate **3** and cytosine provides cytidine 2′-phosphate **5**. (**A**) Synthetic scheme of the coupling of ribose 1,2-cyclic phosphate **3** and cytosine to give cytidine 2′-phosphate **5**. (**B**) HPLC trace of coupling of ribose 1,2-cyclic phosphate **3** and cytosine and its expanded view. (**C**) ^1^H NMR spectrum of the HPLC purified (collected fraction from 8 to 9 min) fraction of the coupling of **3** and cytosine, which shows the existence of cytidine 2′-phosphate **5** as a major component.

However, the coupling reaction of cytosine and ribose 1,2-cyclic phosphate **3** was successful when the reaction temperature was increased and in the presence of Ca^2+^ (Supplementary Fig. S1); in contrast, Mg^2+^ provided a negligible amount of the product (Supplementary Fig. S4). This is quite surprising given the fact that the coupling of threose 1,2-cyclic phosphate and cytosine with Ca^2+^ did not provide any threofuranosylcytosine 2′-phosphate under the same conditions (Supplementary Fig. S24). In detail, when ribose 1,2-cyclic phosphate **3** and cytosine (1:1 to 1:3 ratio) were incubated at 85–125°C in the presence of CaCl_2_, cytidine 2′-phosphate **5** was produced up to ∼7% yield (Table 1, Fig. 2). The coupling was slower at lower temperature (85°C) and gave lower yield (Table 1).

**Table 1.** Formation of Cytidine 2′-Phosphate **5** with Varying Amounts of Cytosine

***3****: Cytosine*	*Temperature (°C)*	*Reaction time (day)*	*Yield of****5****(%)*
1:1	100	1	1.7
		4	2.7
	85	1	0.4
		4	0.8
	125	1	2.8
1:2	100	1	3.5
		4	5.3
	85	1	0.4
		4	1.0
	125	1	5.9
1:3	100	1	3.8
		4	5.4
	125	1	7.7

A mixture of ribose 1,2-cyclic phosphate **3** (5 μL, 15 mM), cytosine (3.75 mM), and calcium chloride (8 μL,15 mM) in aqueous solution was dried and heated. The yield is based on the amount of limiting starting material.

This reaction largely produced the N^1^-product, with minor materials possibly arising from the reaction of the N^4^-exocylic amine group. The identity of the product was confirmed to be canonical cytidine 2′-phosphate **5** by comparing with synthetic cytidine 2′-phosphate (Supplementary synthetic procedure 1) in two different analytical HPLC columns (Supplementary Figs. S2 and S3), by UV spectra, and ^1^H NMR (Fig. 2, Supplementary Fig. S5). Further proof of the structure was obtained by mass spectroscopy (Supplementary Fig. S20).

**FIG. 3.**
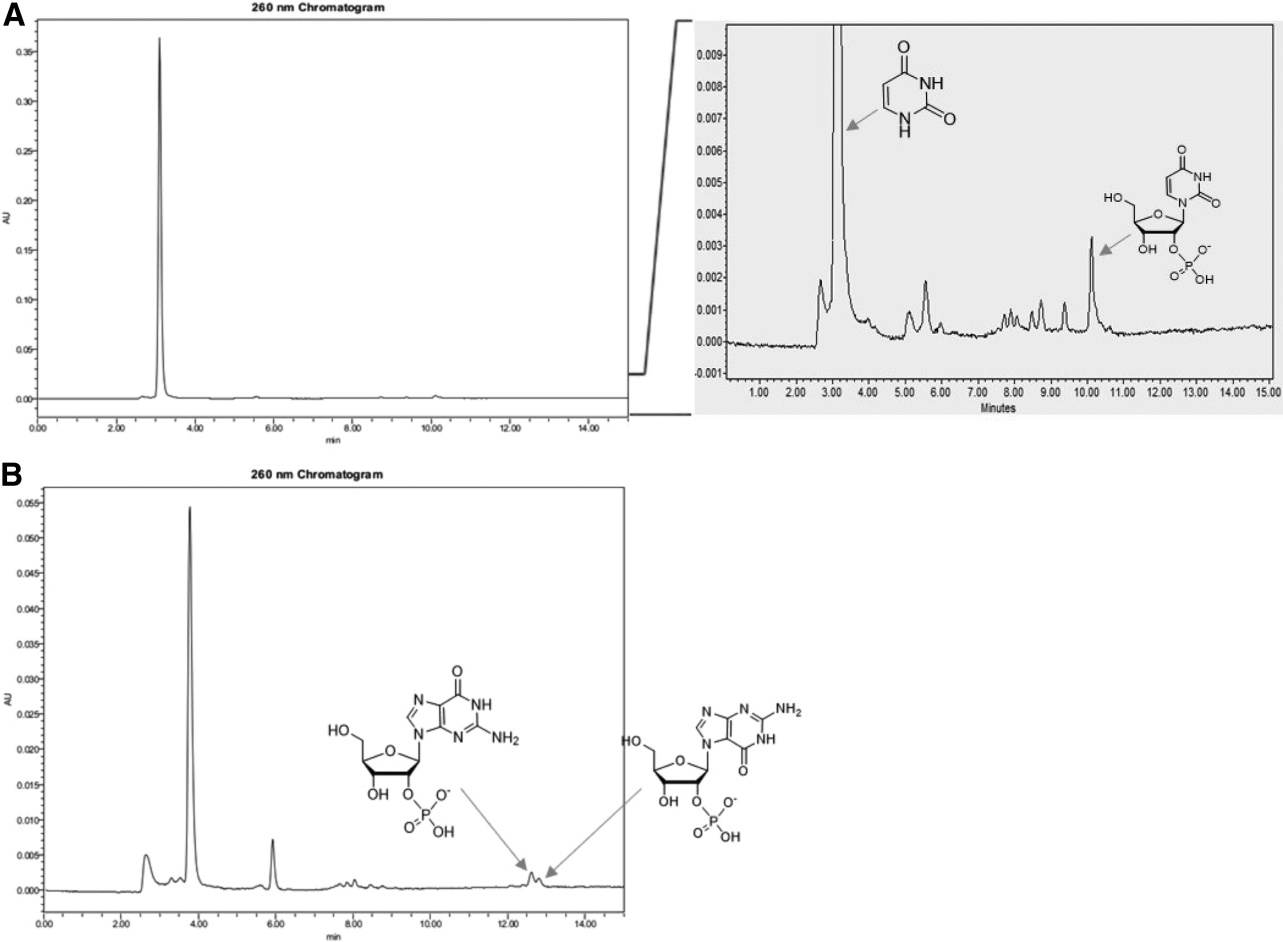
The coupling of ribose 1,2-cyclic phosphate **3** and uracil and guanine provides uridine 2′-phosphate **6** and guanosine 2′-phosphate **7**. (**A**) HPLC trace of coupling of ribose 1,2-cyclic phosphate **3** and uracil and its expanded view to show the formation of uridine 2′-phosphate **6**. (**B**) HPLC trace of coupling of ribose 1,2-cyclic phosphate **3** and guanine and its expanded view to show the formation of guanosine 2′-phosphate **7** (N^9^-ribofuranosylguanine 2′-phosphate) and N^7^-ribofuranosylguanine 2′-phosphate.

Uridine can be made from cytidine by hydrolytic deamination of a cytosine amine group (Powner *et al.,* 2009). However, the direct synthesis of uridine from uracil would also be interesting, as uracil is known in meteorites (Stoks and Schwartz, 1979) and is likely to have been present in relative abundance on a prebiotic Earth, with cytosine, by endogenous synthesis (Robertson and Miller, 1995). The coupling of uracil and ribose 1,2-cyclic phosphate **3** was less efficient than cytosine and required a higher temperature for the coupling reaction. When uracil and ribose 1,2-cyclic phosphate **3** was incubated at 125°C with calcium ion, the reaction mixture gave uridine 2′-phosphate **6** in 0.8% yield (Fig. 3).

Interestingly, these reaction conditions also worked for both canonical purines, adenine, and guanine. The adenosine phosphate from this methodology was reported earlier (Kim and Benner, 2017). Thus, the coupling of guanine and ribose 1,2-cyclic phosphate **3** with Ca^2+^ provided guanosine 2′-phosphate **7**, notwithstanding technical issues due to the insolubility of guanine in water (Fig. 3). For these experiments, the guanine was dissolved in 0.5 M ammonia solution, mixed with ribose 1,2-cyclic phosphate **3**, CaCl_2_, and acetamide. This mixture was evaporated and heated at 125°C for 18 h. Analysis by ion exchange HPLC identified two monophosphated guanosine species in 12–13 min region in HPLC. One was assigned to be guanosine 2′-phosphate **7** (0.34%, N^9^-ribofuranosylguanine 2′-phosphate) by comparison with synthetic material, showing identity by HPLC mobility and UV (Supplementary Figs. S10 and 11) and high-resolution mass spectroscopy (Supplementary Fig. S22). The second compound peak was assigned to be N^7^-ribofuranosylguanine 2′-phosphate (0.74%) initially based on its UV spectrum. Further, N^7^-ribofuranosylguanine was synthesized by the condensation of silylated guanine with tetraacetylated ribose in the presence of SnCl_4_ (Garner and Ramakanth, 1988); comparison of UV spectrum of the synthetic material with the material obtained by the prebiotic synthesis confirmed its structure (Supplementary Fig. S23).

## 4. Discussion

These results show that all four of the glycosidic bonds in the four canonical nucleosides for RNA can be made directly from ribose 1,2-cyclic phosphate **3** and nucleobases, under conditions of intermittently dry, hot (≥100°C), early Earth environments. Such environments are expected in the vicinity of volcanic activity beneath an atmosphere believed to have existed for ∼200 million years following the formation of the late veneer (Genda *et al.,* 2017). It is worth noting that the glycosylation in this study requires the calcium ion, though the phosphorylation of ribose requires the magnesium ion. In the case of glycosylation of adenine, the reaction was successful with either calcium or magnesium (Kim and Benner, 2017). However, in the case of cytosine, the magnesium ion was much worse than that of calcium, and we used calcium through our study for uracil and guanine. This is not the critical problem because both calcium and magnesium are common elements in Earth's crust.

One of the glycosidic bond formations presented in this study (in case of cytosine) gave a generally acceptable reaction yield (∼7%), but some of the others (uracil and guanine) gave a very low condensation yield (less than 1%). The limitations of the current study due to the low yield of uracil and guanine can be mitigated by some justifications. In the case of uracil, although uracil gave a low condensation yield with ribose 1,2-cyclic phosphate, uridine can be generated from cytidine by deamination reaction. This means that the higher yield of condensation of cytosine can be translated to the acceptable synthesis of uridine. In the case of guanine, the prebiotic synthesis of guanosine often provided a low reaction yield (Fuller *et al.,* 1972; Becker *et al.,* 2016). This can be attributed to the very low solubility of guanine.

Besides the reaction yield, all the materials in this study can be obtained under prebiotic conditions. Generation of the nucleobases requires classical reactions of hydrogen cyanide, cyanoacetylene, and other species available from the atmosphere, either directly or indirectly by way of formamide. Generation of the ribose requires atmospheric formaldehyde percolating through serpentinizing basalts that contain igneous borate minerals (*e.g.,* tourmalines). The selective prebiotic synthesis of ribose with high yield has not been reported, although the borate mineral shows selective affinity and stabilization of ribose (Ricardo *et al.,* 2004; Furukawa and Kakegawa, 2017). This may be why some investigators do not start with ribose in their prebiotic synthesis of nucleosides (Powner *et al.,* 2009). However, other successful prebiotic syntheses of nucleosides still require ribose (Becker *et al.,* 2016). These circumstances indicate that the high-yield selective ribose synthesis is needed for the successful prebiotic synthesis of RNA. Generation of the amidotriphosphate requires ammonia (from the atmosphere) to react in water with cyclic trimetaphosphate, which is made by strong dehydration of inorganic phosphate.

## Supplementary Material

Supplemental data

## Abbreviations Used

HPLC: high-performance liquid chromatography
NMR: nuclear magnetic resonance

## References

[B1] Becker S., Thoma I., Deutsch A., Gehrke T., Mayer P., Zipse H., and Carell T. (2016) A high-yielding, strictly regioselective prebiotic purine nucleoside formation pathway. Science 352:833–836 27174989 10.1126/science.aad2808

[B2] Breslow R., Ramalingam V., and Appayee C. (2013) Catalysis of glyceraldehyde synthesis by primary or secondary amino acids under prebiotic conditions as a function of pH. Orig Life Evol Biosph 43:323–329 24346788 10.1007/s11084-013-9347-0

[B3] Fathi R. and Jordan F. (1986) α-D-Ribofuranosyl 1,2-cyclic monophosphate. Isolation, NMR spectroscopic properties, and rates and mechanism of acid and alkaline hydrolysis. J Org Chem 51:4143–4146

[B4] Fuller W.D., Sanchez R.A., and Orgel L.E. (1972) Studies in prebiotic synthesis: VII. Solid-state synthesis of purine nucleosides. J Mol Evol 1:249–257 4681226 10.1007/BF01660244

[B5] Furukawa Y. and Kakegawa T. (2017) Borate and the origin of RNA: a model for the precursors to life. Elements 13:261–265

[B6] Garner P. and Ramakanth S. (1988) A regiocontrolled synthesis of N^7^- and N^9^-guanine nucleosides. J Org Chem 53:1294–1298

[B7] Genda H., Brasser R., and Mojzsis S.J. (2017) The terrestrial late veneer from core disruption of a lunar-sized impactor. Earth Planet Sci Lett 480:25–32

[B8] Gilbert W. (1986) Origin of life: the RNA world. Nature 319:618

[B9] Guerrier-Takada C., Gardiner K., Marsh T., Pace N., and Altman S. (1983) The RNA moiety of ribonuclease P is the catalytic subunit of the enzyme. Cell 35:849–857 6197186 10.1016/0092-8674(83)90117-4

[B10] Harrison T.M., Blichert-Toft J., Müller W., Albarede F., Holden P., and Mojzsis S.J. (2005) Heterogeneous Hadean hafnium: evidence of continental crust at 4.4 to 4.5 Ga. Science 310:1947–1950 16293721 10.1126/science.1117926

[B11] Kim H.-J. and Benner S.A. (2017) Prebiotic stereoselective synthesis of purine and non-canonical pyrimidine nucleotide from nucleobases and phosphorylated carbohydrates. Proc Natl Acad Sci USA 114:11315–11320 29073050 10.1073/pnas.1710778114 PMC5664531

[B12] Kim H.-J. and Benner S.A. (2018) A direct prebiotic synthesis of nicotinamide nucleotide. Chem Eur J 24:581–584 29194806 10.1002/chem.201705394

[B13] Kim H.J., Ricardo A., Illangkoon H.I., Kim M.J., Carrigan M.A., Frye F., and Benner S.A. (2011) Synthesis of carbohydrates in mineral-guided prebiotic cycles. J Am Chem Soc 133:9457–9468 21553892 10.1021/ja201769f

[B14] Kim H.-J., Furukawa Y., Kakegawa T., Bita A., Scorei R., and Benner S.A. (2016) Evaporite borate-containing mineral ensembles make phosphate available and regiospecifically phosphorylate ribonucleosides: borate as a multifaceted problem solver in prebiotic chemistry. Angew Chem Int Ed 55:15816–15820 10.1002/anie.201608001 27862722

[B15] Krishnamurthy R., Guntha S., and Eschenmoser A. (2000) Regioselective α-phosphorylation of aldose in aqueous solution. Angew Chem Int Ed 39:2281–2285 10941064

[B16] Kruger K., Grabowski P.J., Zaug A.J., Sands J., Gottschling D.E., and Cech T.R. (1982) Self-splicing RNA: autoexcision and autocyclization of the ribosomal RNA intervening sequence of *Tetrahymena*. Cell 31:147–157 6297745 10.1016/0092-8674(82)90414-7

[B17] Levy M., Miller S.L., and Oro J. (1999) Production of guanine from NH_4_CN polymerizations. J Mol Evol 49:165–168 10441668 10.1007/pl00006539

[B18] Meinert C., Myrgorodska I., de Marcellus P., Buhse T., Nahon L., Hoffmann S.V., d'Hendecourt L.L.S., and Meierhenrich U.J. (2016) Ribose and related sugars from ultraviolet irradiation of interstellar ice analogs. Science 352:208–212 27124456 10.1126/science.aad8137

[B19] Nam I., Lee J.K., Nam H.G., and Zare R.N. (2017) Abiotic production of sugar phosphates and uridine ribonucleoside in aqueous microdroplets. Proc Natl Acad Sci USA 114:12396–12400 29078402 10.1073/pnas.1714896114 PMC5703324

[B20] Nam I., Nam H.G., and Zare R.N. (2018) Abiotic synthesis of purine and pyrimidine ribonucleosides in aqueous microdroplets. Proc Natl Acad Sci USA 115:36–40 29255025 10.1073/pnas.1718559115 PMC5776833

[B21] Orgel L.E. (2004) Prebiotic chemistry and the origin of the RNA world. Crit Rev Biochem Mol Biol 39:99–123 15217990 10.1080/10409230490460765

[B22] Oro J. (1961) Mechanism of synthesis of adenine from hydrogen cyanide under possible primitive Earth conditions. Nature 191:1193–1194 13731264 10.1038/1911193a0

[B23] Pasek M.A., Kee T.P., Bryant D.E., Pavlov A.A., and Lunine J.I. (2008) Production of potentially prebiotic condensed phosphates by phosphorus redox chemistry. Angew Chem Int Ed 47:7918–7920 10.1002/anie.200802145 18781567

[B24] Powner M.W. and Sutherland J.D. (2008) Potentially prebiotic synthesis of pyrimidine β-D-ribonucleotides by photoanomerization/hydrolysis of α-D-cytidine-2′-phosphate. ChemBioChem 9:2386–2387 18798212 10.1002/cbic.200800391

[B25] Powner M.W., Gerland B., and Sutherland J.D. (2009) Synthesis of activated pyrimidine ribonucleotides in prebiotically plausible conditions. Nature 459:239–242 19444213 10.1038/nature08013

[B26] Ricardo A., Carrigan M.A., Olcott A.N., and Benner S.A. (2004) Borate minerals stabilize ribose. Science 303:196 14716004 10.1126/science.1092464

[B27] Robertson M.P. and Miller S.L. (1995) An efficient prebiotic synthesis of cytosine and uracil. Nature 373:772–774 10.1038/375772a0 7596408

[B28] Ruiz-Mirazo K., Briones C., and de la Escosura A. (2014) Prebiotic systems chemistry: new perspectives for the origins of life. Chem Rev 114:285–366 24171674 10.1021/cr2004844

[B29] Saladino R., Carota E., Botta G., Kapralov M., Timoshenko G.N., Rozanov A.Y., Krasavin E., and Di Mauro E. (2015) Meteorite-catalyzed syntheses of nucleosides and of other prebiotic compounds from formamide under proton irradiation. Proc Natl Acad Sci USA 112:E2746–E2755 25870268 10.1073/pnas.1422225112 PMC4450408

[B30] Stairs S., Nikmal A., Bucar D.-K., Zheng S.-L., Szostak J.W., and Powner M.W. (2017) Divergent prebiotic synthesis of pyrimidine and 8-oxo-purine ribonucleotides. Nat Commun 8:15270 28524845 10.1038/ncomms15270 PMC5454461

[B31] Steer A.M., Bia N., Smith D.K., and Clarke P.A. (2017) Prebiotic synthesis of 2-deoxy-D-ribose from interstellar building blocks promoted by amino esters or amino nitriles. Chem Commun 53:10362–10365 10.1039/c7cc06083a 28884758

[B32] Stoks P.G. and Schwartz A.W. (1979) Uracil in carbonaceous meteorites. Nature 282:709–710

[B33] Vorbrüggen H. and Ruh-Pohlenz C. (2000) Synthesis of nucleosides. Org React 55:1–111

[B34] Xu J., Tsanakopoulou M., Magnani C.J., Szabla R., Šponer J.E., Šponer J., Góra R.W., and Sutherland J.D. (2017) A prebiotically plausible synthesis of pyrimidine β-ribonucleosides and their phosphate derivatives involving photoanomerization. Nat Chem 9:303–309 28338689 10.1038/nchem.2664 PMC5576532

