# Supplementary material for: A Prebiotic Synthesis of Canonical Pyrimidine and Purine Ribonucleotides
Source: Astrobiology. 2019 Apr 25;19(5):669–74. doi: 10.1089/ast.2018.1935 (PMC6486662; doi:10.1089/ast.2018.1935)
Supplement: Supplemental data [file Supp_Data.pdf]

Supplementary Materials for

A Prebiotic Synthesis of Canonical Pyrimidine and Purine Ribonucleotides

Hyo-Joong Kim[1*], Justin Kim[2]

[1]Firebird Biomolecular Sciences LLC, Alachua, FL 32615

[2]Buchholz High School, Gainesville, FL 32606

*Correspondence to: hkim@firebirdbio.com

Synthetic procedure

1. Chemical synthesis of cytidine 2'-phosphate (5)

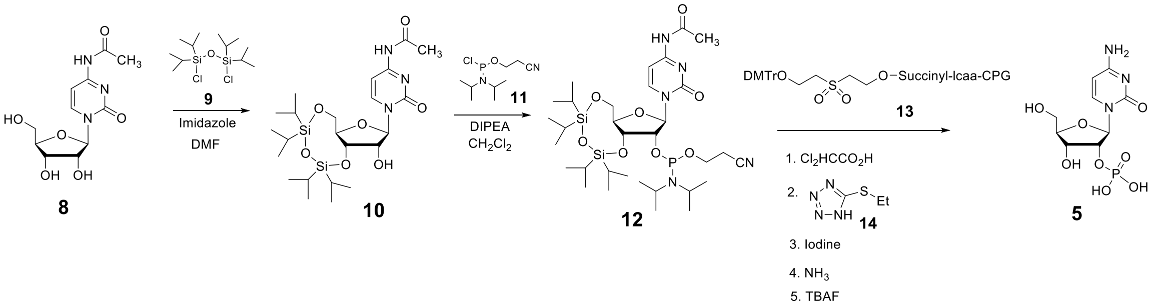

1.1. Synthesis of **10**

To a mixture of N^4-acetylcytidine **8** (500 mg, 1.75 mmol, Carbosynth) and imidazole (480 mg, 7.0 mmol) in N,N-dibutylformamide (6 mL) was added 1,3-dichloro-1,1,3,3-tetraisopropyldisiloxane **9** (670 mg, 2.1 mmol) at room temperature. The mixture was stirred at room temperature for 1 hour and mixed with water (30 mL). The product was extracted with ethyl acetate. The combined organic phase was dried over sodium sulfate and volatiles were removed by rotary evaporation and the residue was purified on silica column (ethyl acetate, R_f=0.5) to give a white solid (570 mg, 1.08 mmol, 62%). ^{1}H NMR (300 MHz, $CDCl_3$) δ 10.48 (br s, 1H), 8.19 (d, 1H, J = 7.5 Hz), 7.42 (d, 1H, J = 7.5 Hz), 5.78 (s, 1H), 3.90~4.30 (m, 5H), 3.71 (br s, 1H), 2.27 (s, 3H), 0.85~1.10 (m, 28H). ^{13}C NMR (75 MHz, $CDCl_3$) δ 171.47, 163.53, 155.30, 144.49, 96.89, 91.93, 82.21, 75.35, 68.85, 60.32, 25.06, 17.56, 17.47, 17.15, 17.07, 16.99, 13.56, 13.17, 12.71.

1.2. Synthesis of **12**

To a mixture of compound **10** (400 mg, 0.76 mmol) and 4-methylmorpholine (0.27 mL, 1.9 mmol) in dichloromethane (10 mL) was added 2-cyanoethyl N,N-diisopropylchloro-phosphoramidite **11** (269 mg, 1.14 mmol) at room temperature. The mixture was stirred at room temperature for 2 hours and mixed with water (30 mL). The product was extracted with dichloromethae. The combined organic phase was dried over sodium sulfate and volatiles were removed by rotary evaporation and the residue was purified on neutral silica column (ethyl acetate:Hexane=1:1 to ethyl acetate:Hexane=4:1) to give a white solid (316 mg, 0.434 mmol, 57%). ^{1}H NMR (300 MHz, $CDCl_3$) δ 8.99 and 8.94 (singlet each, 1H), 8.3~8.4 (m, 1H), 7.35~7.40 (m, 1H), 5.80 and 5.85 (singlet each, 1H), 3.6~4.4 (m, 9H), 2.6~2.9 (m, 2H), 2.23 (s, 3H), 0.9~1.3 (m, 40H). ^{31}P NMR (121 MHz, $CDCl_3$) δ 154.35, 152.17.

1.3. Synthesis of **5**

Cytidine 2'-phosphate **5** was synthesized from **12** on DNA synthesizer (ABI 394). Dimethoxytrityl was removed from 3'-phosphate CPG **13** (26 mg, 1 μmole, Glen Research) by dichloroacetic acid. To the CPG **13** was added an acetonitrile solution of **12** (0.1M) and coupled by the addition of thioethyl tetrazole **14**. Phosphorus (III) was oxidized by iodine to phosphorus (V). The CPG was dried and mixed with ammonium hydroxide (0.3 mL). The mixture was heated at 55 °C for 1 hour. After cooling to room temperature the liquid was separated from the CPG and the CPG was washed with a mixture of water/acetonitrile (1:1). The liquid and washing were combined and

lyophilized then treated with acetonitrile (0.5 mL) and 1M tetrabutylammonium fluoride (0.05 mL). The mixture was shaken at room temperature for 2 hours and diluted with water (5 mL) and purified by ion exchange prep HPLC. Preparative HPLC purification was done using an ion exchange column (22 mm id, 250 mm length, 5 µm; DNAPac PA-100; Thermo Fisher Scientific) on a Waters Delta 600 module. The column was eluted with a gradient of (A) water and (B) 1M ammonium bicarbonate. The elution program created a linear gradient started from 100% A to 80% A at 15 min with flow rate of 10 ml/min. Peak detection was conducted using the 260 nm absorbance. The appropriate fractions were collected and lyophilized to give **5**. **^{1}H-NMR** (300 MHz, D_2O) δ = 7.64 (d, 1H, *J* = 7.8 Hz), 5.90 (d, 1H, *J* = 7.8 Hz), 5.83 (d, 1H, *J* = 4.8 Hz), 4.19 (m, 1H), 3.96 (m, 1H), 3.5~3.8 (m, 3H). **HRMS** (ESI-): calc. for: : $[C_9H_{13}N_3O_8P]^-$ 322.0446, found: 322.0458 $[M-H]^-$.

2. Chemical synthesis of uridine 2'-phosphate (6)

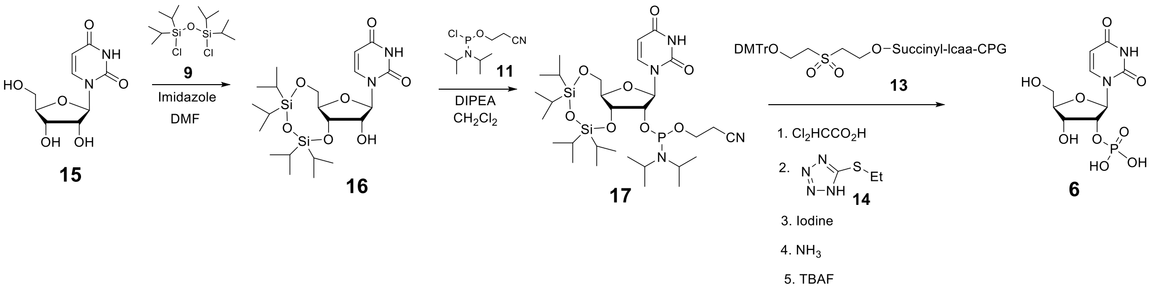

2.1. Synthesis of **16**

To a mixture of uridine **15** (1.00 g, 4.1 mmol, Alfa Aesar) and imidazole (1.12 g, 16.4 mmol) in N,N-dibutylformamide (10 mL) was added 1,3-dichloro-1,1,3,3-tetraisopropyldisiloxane **9** (1.55 g, 4.9 mmol) at room temperature. The mixture was stirred at room temperature for 1 hour and mixed with water (50 mL). The product was extracted with ethyl acetate. The combined organic phase was dried over sodium sulfate and volatiles were removed by rotary evaporation and the residue was purified on silica column (ethyl acetate:hexane=2:1, R_f=0.5) to give a white solid (1.53 g, 3.14 mmol, 77%). ^{1}H NMR (300 MHz, $CDCl_3$) δ 9.41 (br s, 1H), 7.74 (d, 1H, *J* = 8.1 Hz), 5.73 (s, 1H), 5.69 (d, 1H, *J* = 8.1 Hz), 3.90~4.40 (m, 5H), 3.48 (s, 1H), 0.95~1.10 (m, 28H). ^{13}C NMR (75 MHz, $CDCl_3$) δ 163.28, 150.15, 140.23, 102.16, 91.29, 82.22, 75.38, 69.43, 60.70,17.45, 17.22, 13.60, 13.16, 12.79.

2.2. Synthesis of **17**

To a mixture of compound **16** (700 mg, 1.44 mmol) and 4-methylmorpholine (0.50 mL, 3.6 mmol) in dichloromethane (15 mL) was added 2-cyanoethyl N,N-diisopropylchloro-phosphoramidite **11** (510 mg, 2.16 mmol) at room temperature. The mixture was stirred at room temperature for 2 hours and mixed with water (50 mL). The product was extracted with dichloromethae. The combined organic phase was dried over sodium sulfate and volatiles were removed by rotary evaporation and the residue was purified on neutral silica column (ethyl acetate:Hexane=1:2 to ethyl acetate:hexane=1:1) to give a

white solid (570 mg, 0.83 mmol, 58%). ^{1}H NMR (300 MHz, $CDCl_3$) δ 8.45 and 8.67 (br singlet each, 1H), 7.93 (d, 1H, *J* = 8.4 Hz), 5.81 and 5.75 (singlet each, 1H), 5.66 (d, 1H, *J* = 8.4 Hz), 3.6~4.4 (m, 9H), 2.6~2.8 (m, 2H), 0.9~1.3 (m, 40H). ^{31}P NMR (121 MHz, $CDCl_3$) δ 154.27, 152.39.

2.3. Synthesis of **6**

Uridine 2'-phosphate **6** was synthesized from **17** on DNA synthesizer (ABI 394). Dimethoxytrityl was removed from 3'-phosphate CPG **13** (26 mg, 1 µmole, Glen Research) by dichloroacetic acid. To the CPG **13** was added an acetonitrile solution of **17** (0.1M) and coupled by the addition of thioethyl tetrazole **14**. Phosphorus (III) was oxidized by iodine to phosphorus (V). The CPG was dried and mixed with ammonium hydroxide (0.3 mL). The mixture was heated at 55 °C for 1 hour. After cooling to room temperature the liquid was separated from the CPG and the CPG was washed with a mixture of water/acetonitrile (1:1). The liquid and washing were combined and lyophilized then treated with acetonitrile (0.5 mL) and 1M tetrabutylammonium fluoride (0.05 mL). The mixture was shaken at room temperature for 2 hours and diluted with water (5 mL) and purified by ion exchange prep HPLC. Preparative HPLC purification was done using an ion exchange column (22 mm id, 250 mm length, 5 µm; DNAPac PA-100; Thermo Fisher Scientific) on a Waters Delta 600 module. The column was eluted with a gradient of (A) water and (B) 1M ammonium bicarbonate. The elution program created a linear gradient started from 100% A to 80% A at 15 min with flow rate of 10 ml/min. Peak detection was conducted using the 260 nm absorbance. The appropriate fractions were collected and lyophilized to give **6**. **^{1}H-NMR** (300 MHz, D_2O) δ = 7.65 (d, 1H, *J* = 8.1 Hz), 5.84 (d, 1H, *J* = 5.7 Hz), 5.73 (d, 1H, *J* = 8.4 Hz), 4.19 (m, 1H), 3.97 (m, 1H), 3.6~3.85 (m, 3H). **HRMS** (ESI-): calc. for: $[C_9H_{12}N_2O_9P]^-$ 323.0286, found: 323.0290 $[M-H]^-$.

3. Chemical synthesis of guanosine 2'-phosphate (7)

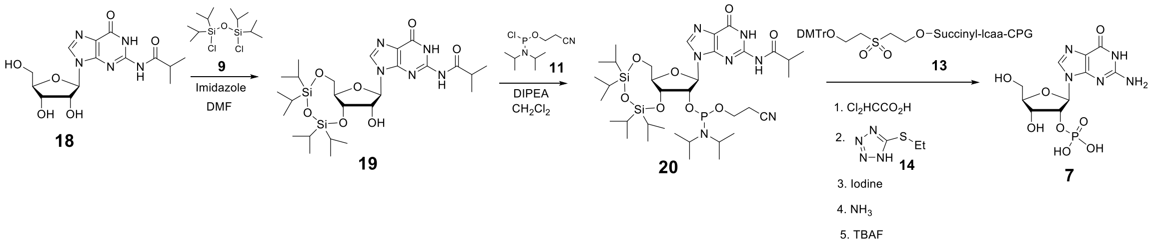

3.1. Synthesis of **19**

To a mixture of N^2-isobutyrylguanosine **18** (1.45 g, 4.1 mmol, Carbosynth) and imidazole (1.12 g, 16.4 mmol) in N,N-dibutylformamide (10 mL) was added 1,3-dichloro-1,1,3,3-tetraisopropyldisiloxane **9** (1.55 g, 4.9 mmol) at room temperature. The mixture was stirred at room temperature for 1 hour and mixed with water (50 mL). The product was extracted with ethyl acetate. The combined organic phase was dried over sodium sulfate and volatiles were removed by rotary evaporation and the residue was purified on silica column (ethyl acetate, R_f=0.6) to give a white solid (1.55 g, 2.6 mmol, 63%). ^{1}H NMR (300 MHz, $CDCl_3$) δ 12.14 (s, 1H), 9.49 (s, 1H), 7.92 (s, 1H), 5.85 (s, 1H), 3.95~4.55 (m, 5H), 3.63 (s, 1H), 2.75 (Septet, 1H, *J* = 6.9 Hz), 0.8~1.3 (m, 34H). ^{13}C NMR (75 MHz,

$CDCl_3$) δ 178.90, 155.75, 147.97, 147.77, 136.85, 121.91, 89.06, 82.14, 75.52, 70.35, 61.34, 36.67, 19.18, 19.11, 17.59, 17.43, 17.28, 17.15, 17.05, 13.58, 13.21, 12.84.

3.2. Synthesis of **20**

To a mixture of compound **19** (858 mg, 1.44 mmol) and 4-methylmorpholine (0.50 mL, 3.6 mmol) in dichloromethane (15 mL) was added 2-cyanoethyl N,N-diisopropylchloro-phosphoramidite **11** (510 mg, 2.16 mmol) at room temperature. The mixture was stirred at room temperature for 2 hours and mixed with water (50 mL). The product was extracted with dichloromethae. The combined organic phase was dried over sodium sulfate and volatiles were removed by rotary evaporation and the residue was purified on neutral silica column (dichloromethane to dichloromethane:methanol=20:1) to give a white solid (320 mg, 0.40 mmol, 28%). ^{1}H NMR (300 MHz, $CDCl_3$) δ 12.13 (br s, 1H), 9.34 (s, 1H), 8.18 (s, 1H), 5.99 (s, 1H), 3.6~4.6 (m, 9H), 2.5~2.8 (m, 3H), 0.9~1.3 (m, 46H). ^{31}P NMR (121 MHz, $CDCl_3$) δ 152.54, 150.21.

3.3. Synthesis of **7**

Guanosine 2'-phosphate **7** was synthesized from **20** on DNA synthesizer (ABI 394). Dimethoxytrityl was removed from 3'-phosphate CPG **13** (26 mg, 1 μmole, Glen Research) by dichloroacetic acid. To the CPG **13** was added an acetonitrile solution of **20** (0.1M) and coupled by the addition of thioethyl tetrazole **14**. Phosphorus (III) was oxidized by iodine to phosphorus (V). The CPG was dried and mixed with ammonium hydroxide (0.3 mL). The mixture was heated at 55 °C for 4 hours. After cooling to room temperature the liquid was separated from the CPG and the CPG was washed with a mixture of water/acetonitrile (1:1). The liquid and washing were combined and lyophilized then treated with acetonitrile (0.5 mL) and 1M tetrabutylammonium fluoride (0.05 mL). The mixture was shaken at room temperature for 2 hours and diluted with water (5 mL) and purified by ion exchange prep HPLC. Preparative HPLC purification was done using an ion exchange column (22 mm id, 250 mm length, 5 μm; DNAPac PA-100; Thermo Fisher Scientific) on a Waters Delta 600 module. The column was eluted with a gradient of (A) water and (B) 1M ammonium bicarbonate. The elution program created a linear gradient started from 100% A to 80% A at 15 min with flow rate of 10 ml/min. Peak detection was conducted using the 260 nm absorbance. The appropriate fractions were collected and lyophilized to give **7**. **^{1}H-NMR** (300 MHz, D_2O) δ = 7.83 (s, 1H), 5.86 (d, 1H, J = 6.0 Hz), 4.97 (m, 1H), 4.37 (m, 1H), 4.09 (m, 1H), 3.6~3.8 (m, 2H). **HRMS** (ESI-): calc. for: $[C_{10}H_{13}N_5O_8P]^-$ 362.0496, found: 362.0508 $[M-H]^-$

4. Synthesis of N^7-ribofuranosylguanine (25)

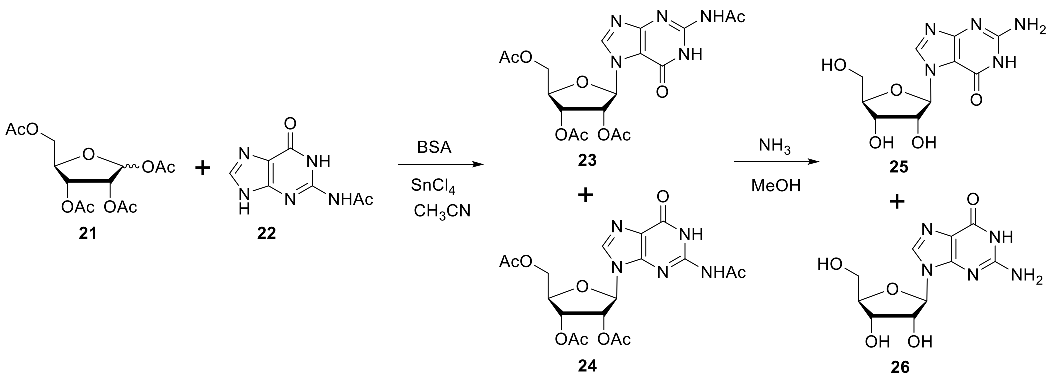

The N[7]-ribofuranosylguanine **25** was synthesized following the reported method (P. Garner, S. Ramakanth, A regiocontrolled synthesis of N[7]- and N[9]-guanine nucleosides. *J. Org. Chem.* **53**, 1294-1298 (1988)). To a suspension of the tetraacetylated ribose **21** (318 mg, 1.0 mmol) and 2-acetylguanine **22** (193 mg, 1.0 mmol) in acetonitrile (5 mL) was added N,O-bis(trimethylsilyl) acetamide (0.61 mL) and heated to 70 °C. Stirring and heating was continued until a clear solution was formed (1 hour). The mixture was cooled to room temperature and $SnCl_4$ (1 M solution in CH_2Cl_2, 3.0 mL) was added and stirred at room temperature for 3 hours. The mixture was poured into water and extracted with dichloromethane. The combined organic phase was dried over sodium sulfate and evaporated. The residue was purified on silica column (ethyl acetate:methanol=20:1) to give a white solid (158 mg, 35%) as a mixture of **23**/**24**. To the solid (10 mg) was added methanol (0.1 mL) and ammonium hydroxide (0.2 mL) and the mixture was heated at 55 °C for 18 hours. Ammonia was removed from the mixture by evaporation and the residue was analyzed by reverse phase HPLC to give a mixture of N[7]- and N[9]-ribofuranosylguanine (Fig S21).

5. Attempted synthesis of threofuranosylcytosine 2'-phosphate (28) by the coupling of threose 1,2-cyclic phosphate (27) and cytosine

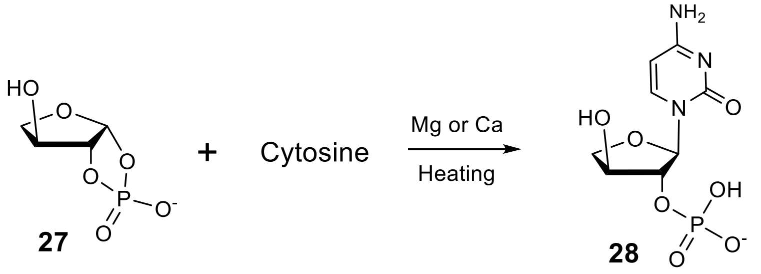

The reaction of **27** and cytosine was conducted in an Eppendorf tube containing threose 1,2-cyclic phosphate **27** (5 µL, 15 mM), cytosine (10 µL, 3.75 mM) and calcium chloride (7.5 µL,15 mM) or magnesium chloride (7.5 µL,15 mM) in aqueous solution. The mixture was dried and heated at 100 °C for 1 day. It was re-dissolved in water (0.3 mL) and analyzed by ion exchange HPLC. The analysis of the reaction product showed no formation of **28**.

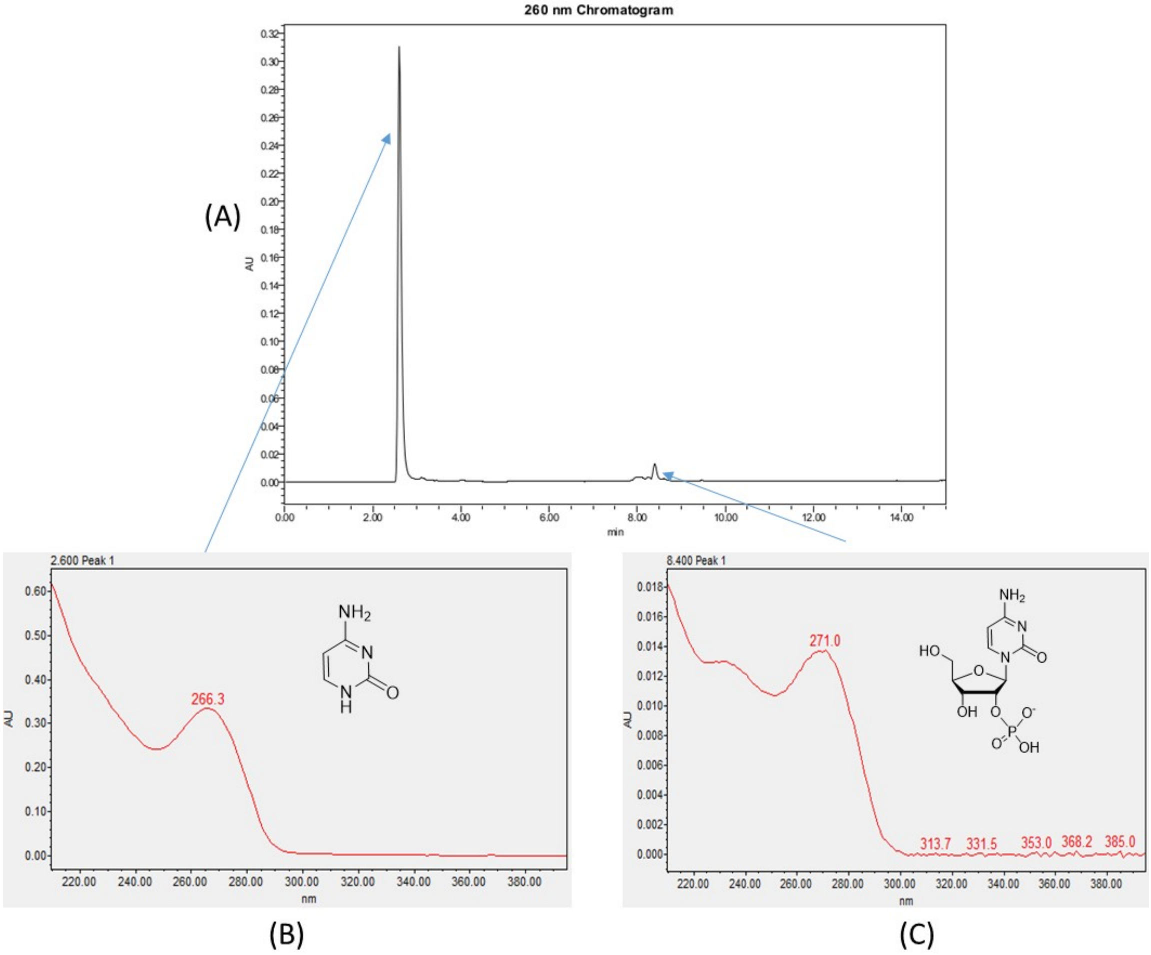

Fig. S1.

Formation of cytidine 2'-phosphate 5. (A) HPLC trace of the formation of cytidine 2'-phosphate **5** from the coupling of ribose 1,2-cyclic phosphate **3** and cytosine**.** (B) Extracted UV spectrum of the peak at 2.6 min that showed absorption maximum at 266 nm. This UV is identical to cytosine. (C) Extracted UV spectrum of the peak at 8.3 min that showed absorption maximum at 270 nm. This UV is identical to synthetic cytidine 2'-phosphate.

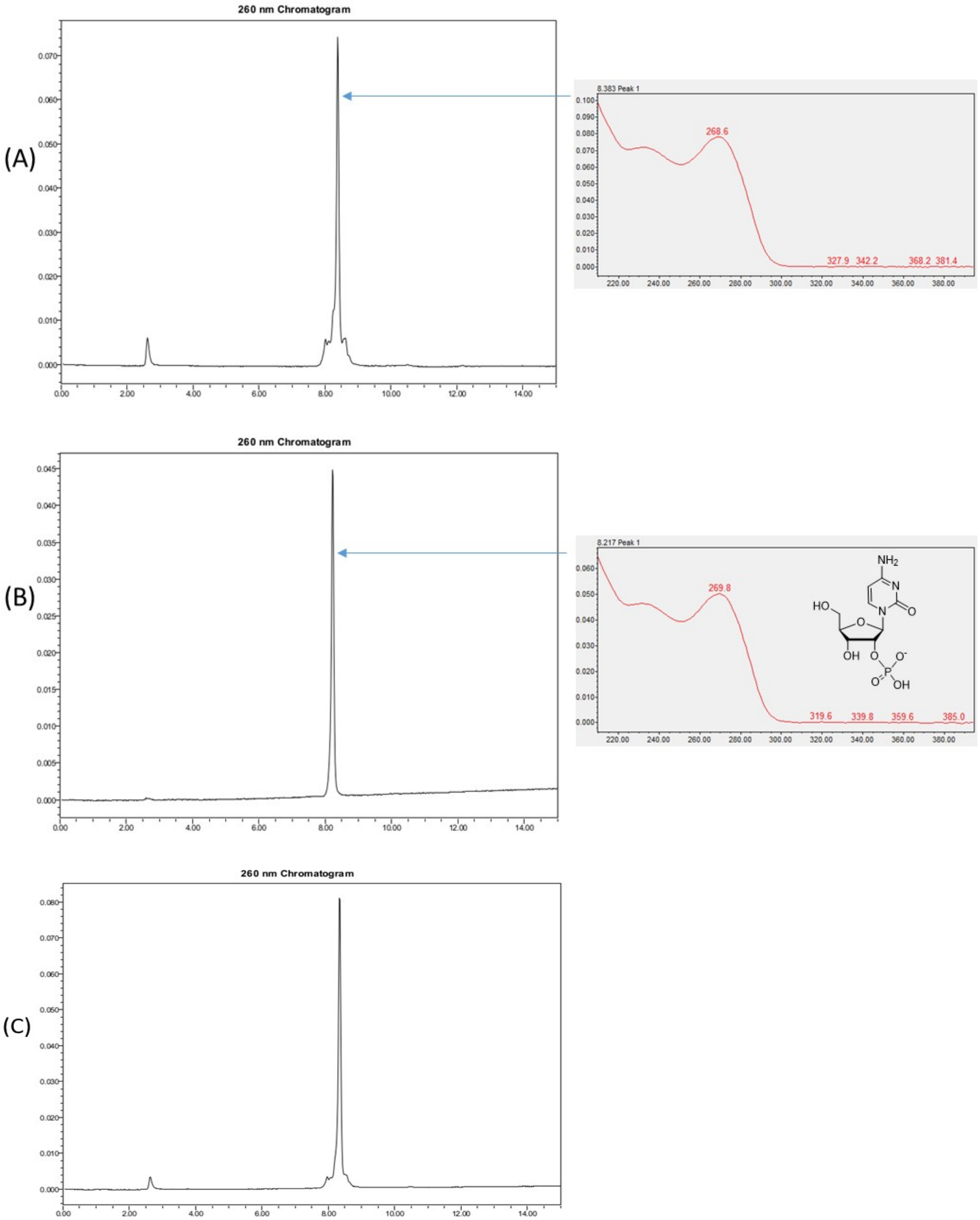

Fig. S2.

Formation of cytidine 2'-phosphate 5. (A) Ion exchange HPLC trace of the purified fraction (collection around 8.3 min) of the coupling of ribose 1,2-cyclic phosphate **3** and cytosine and its UV spectrum**.** HPLC shows one major peak and many small peaks around. (B) Ion exchange HPLC trace of cytidine 2'-phosphate by chemical synthesis and its UV spectrum. (C) HPLC trace of co-injection of (A) and (B) which confirms (A) and (B) are same compound.

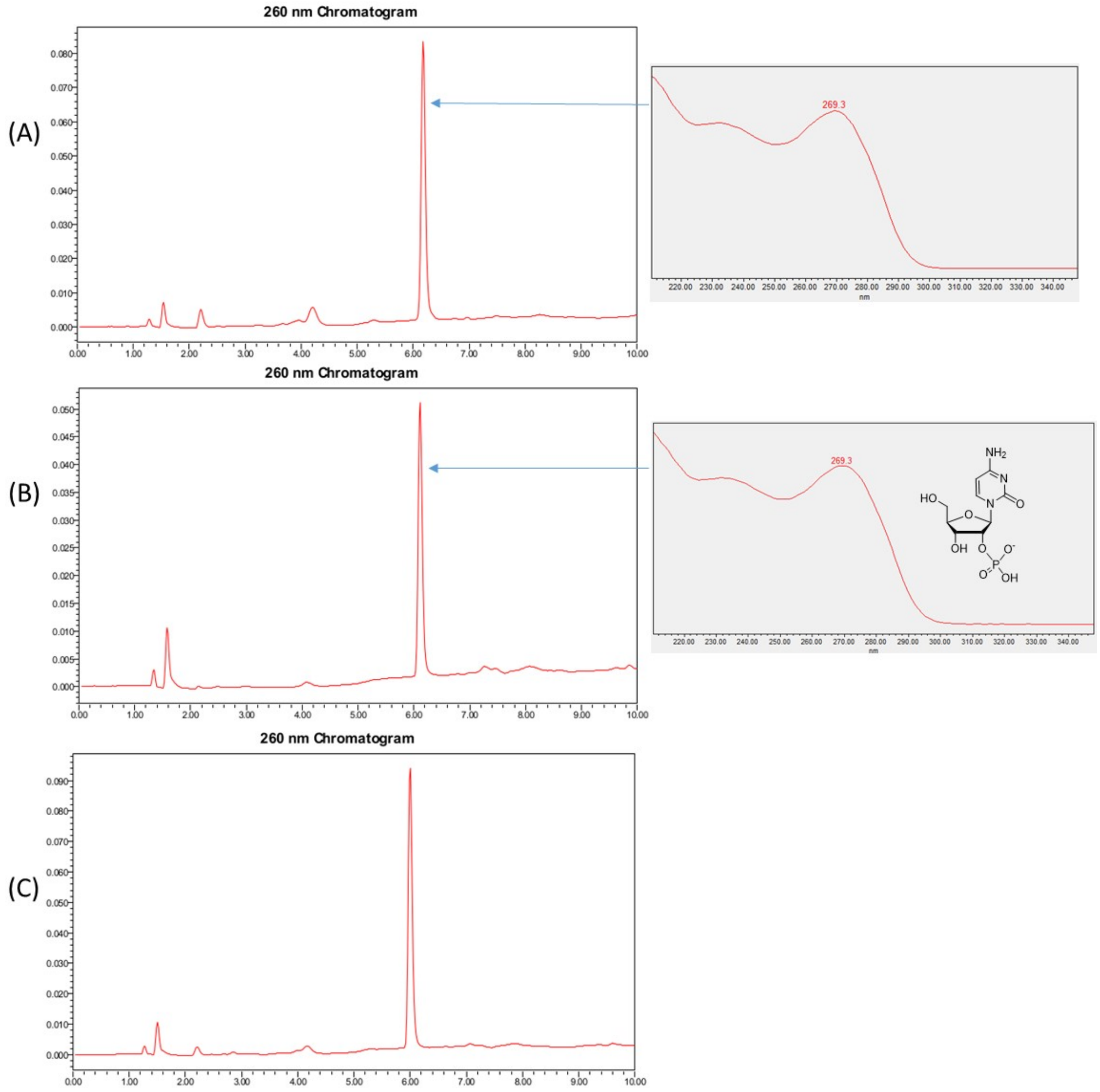

Fig. S3.

Formation of cytidine 2'-phosphate 5. (A) Reverse phase HPLC trace of the purified fraction (collection around 8.3 min of ion exchange HPLC) of the coupling of ribose 1,2-cyclic phosphate **3** and cytosine and its UV spectrum**.** HPLC shows one major peak at 6.2 min. (B) Reverse phase HPLC trace of cytidine 2'-phosphate by chemical synthesis and its UV spectrum. (C) HPLC trace of co-injection of (A) and (B) which confirms (A) and (B) are same compound.

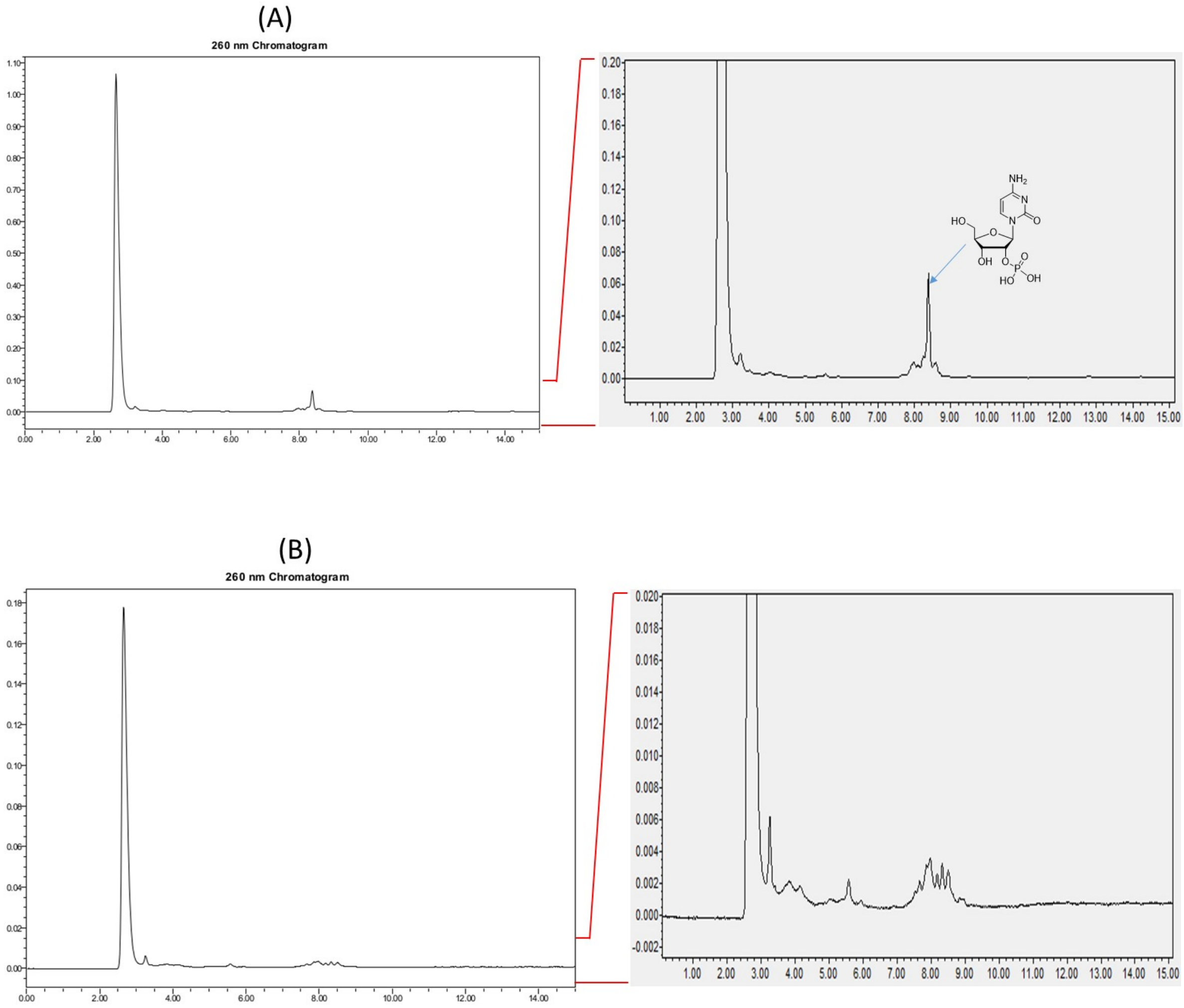

Fig. S4.

Formation of cytidine 2’-phosphate 5. An aqueous mixture of cytosine and ribose 1,2-cyclic phosphate was dried and heated at 125 °C for 1 day in the presence of Ca^{2+} or Mg^{2+}. (A) Ion exchange HPLC trace of the coupling of ribose 1,2-cyclic phosphate **3** and cytosine with Ca^{2+} and its expanded HPLC trace which shows the formation of cytidine 2’-phosphate. (B) Ion exchange HPLC trace of the coupling of ribose 1,2-cyclic phosphate **3** and cytosine with Mg^{2+} and its expanded HPLC trace which shows the formation of cytidine 2’-phosphate is not clear.

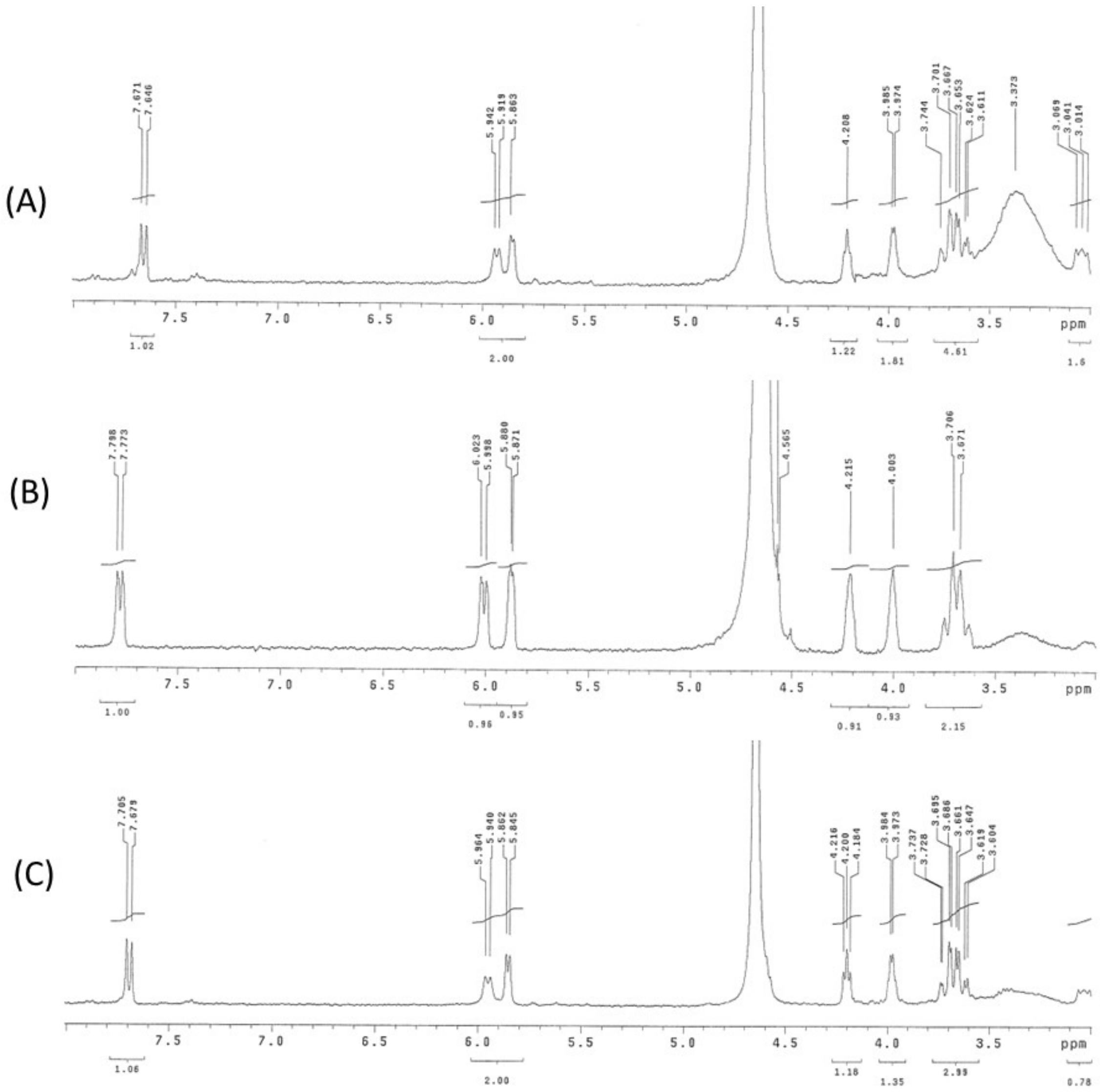

Fig. S5

^{1}H NMR spectrum of cytidine 2'-phosphate 5. (A) ^{1}H NMR spectrum of the purified fraction of the coupling of ribose 1,2-cyclic phosphate **3** and cytosine. The NMR shows cytidine 2'-phosphate peak as major NMR signals along with other smaller peaks. (B) ^{1}H NMR spectrum of chemically synthesized cytidine 2'-phosphate. (C) ^{1}H NMR spectrum of the mixture of (A) and (B) which shows the increase the intensity of the peaks of cytidine 2'-phosphate.

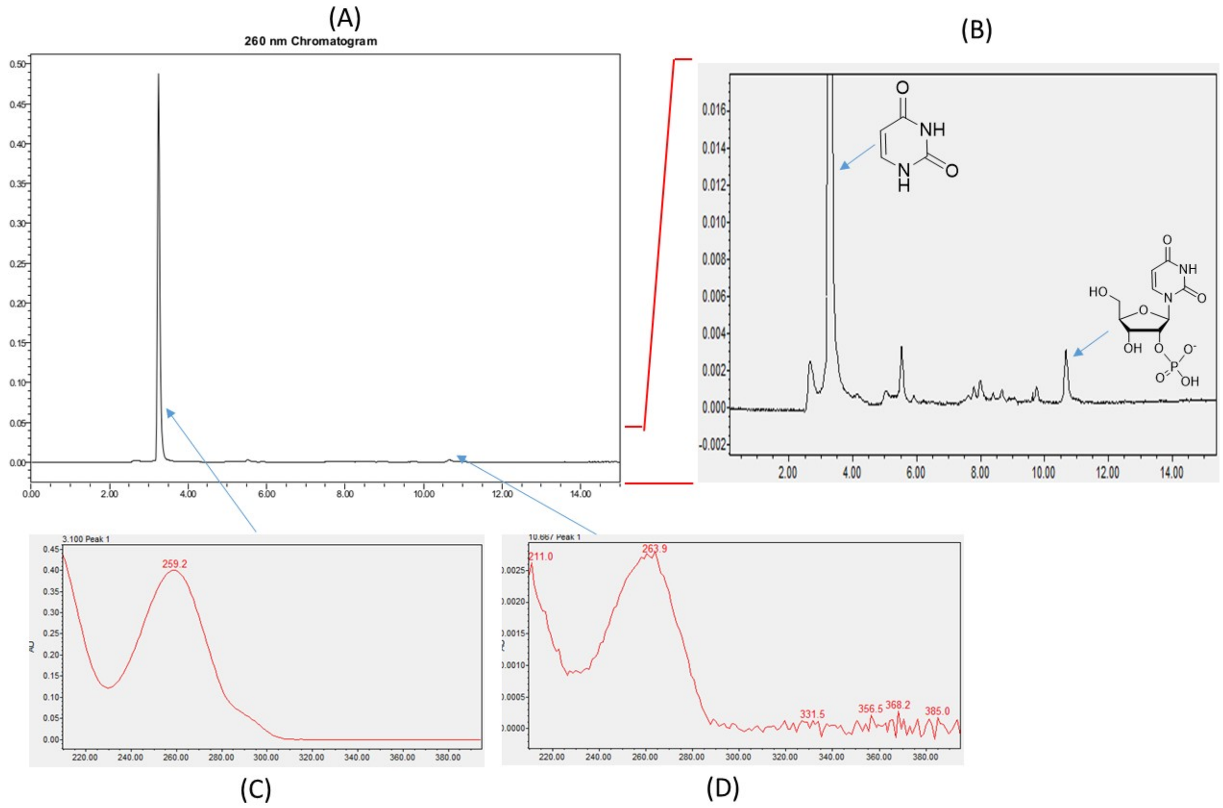

Fig. S6.

Formation of uridine 2’-phosphate 6. (A) HPLC trace of the formation of uridine 2’-phosphate **6** from the coupling of ribose 1,2-cyclic phosphate **3** and uracil**.** (B) Expanded HPLC trace of (A). (C) Extracted UV spectrum of the peak at 3.1 min that showed absorption maximum at 259 nm. This UV is identical to uracil. (D) Extracted UV spectrum of the peak at 10.7 min that showed absorption maximum at 261 nm. This UV is identical to synthetic uridine 2’-phosphate.

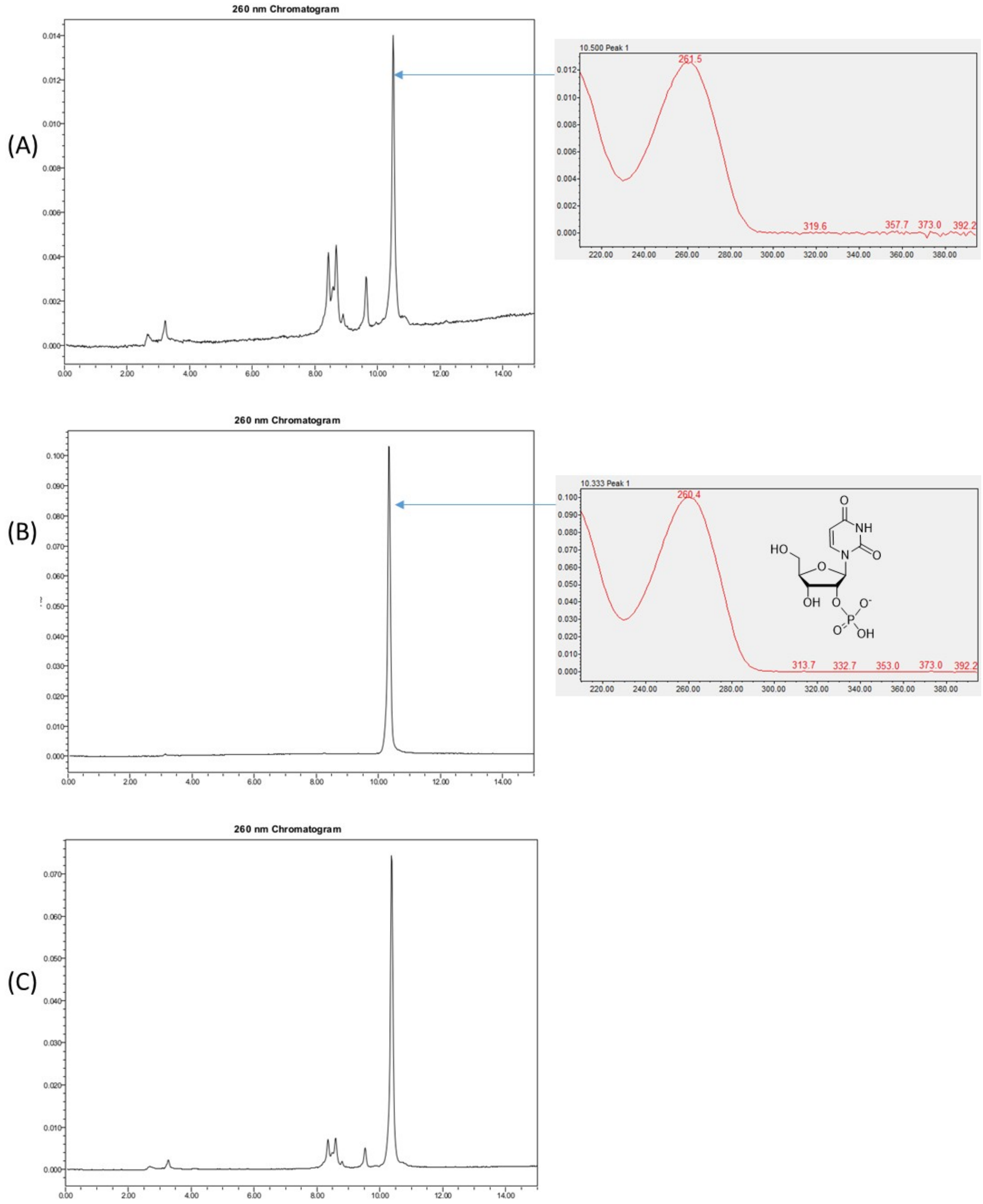

Fig. S7.

Formation of uridine 2'-phosphate 6. (A) Ion exchange HPLC trace of the purified fraction (collection around 10.7 min) of the coupling of ribose 1,2-cyclic phosphate **3** and uracil and its UV spectrum**.** HPLC shows one major peak. (B) Ion exchange HPLC trace of uridine 2'-phosphate by chemical synthesis and its UV spectrum. (C) HPLC trace of co-injection of (A) and (B) which confirms (A) and (B) are same compound.

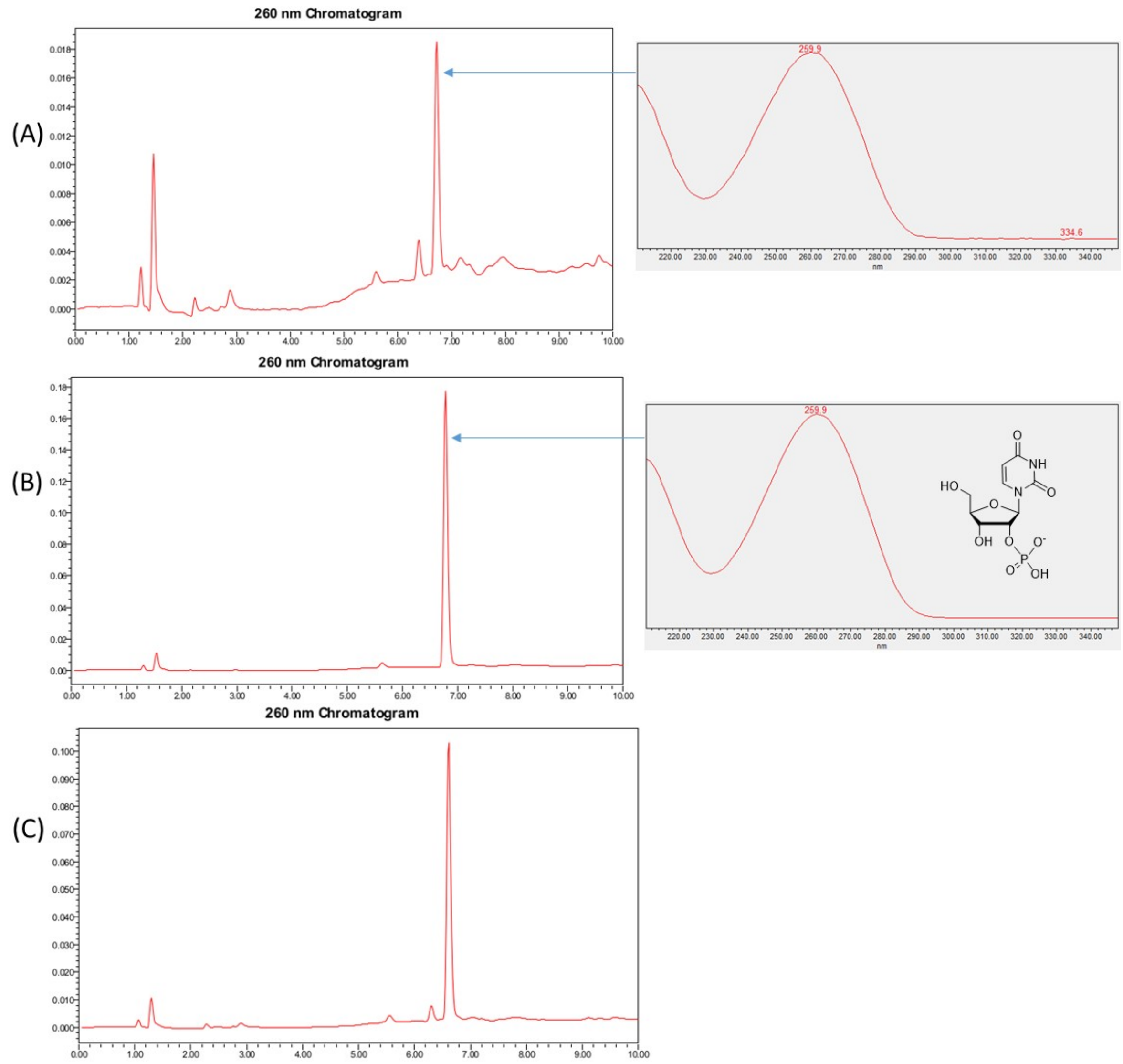

Fig. S8.

Formation of uridine 2'-phosphate 6. (A) Reverse phase HPLC trace of the purified fraction (collection around 10.7 min of ion exchange HPLC) of the coupling of ribose 1,2-cyclic phosphate **3** and uracil and its UV spectrum**.** HPLC shows one major peak at 6.8 min. (B) Reverse phase HPLC trace of uracil 2'-phosphate by chemical synthesis and its UV spectrum. (C) HPLC trace of co-injection of (A) and (B) which confirms (A) and (B) are same compound.

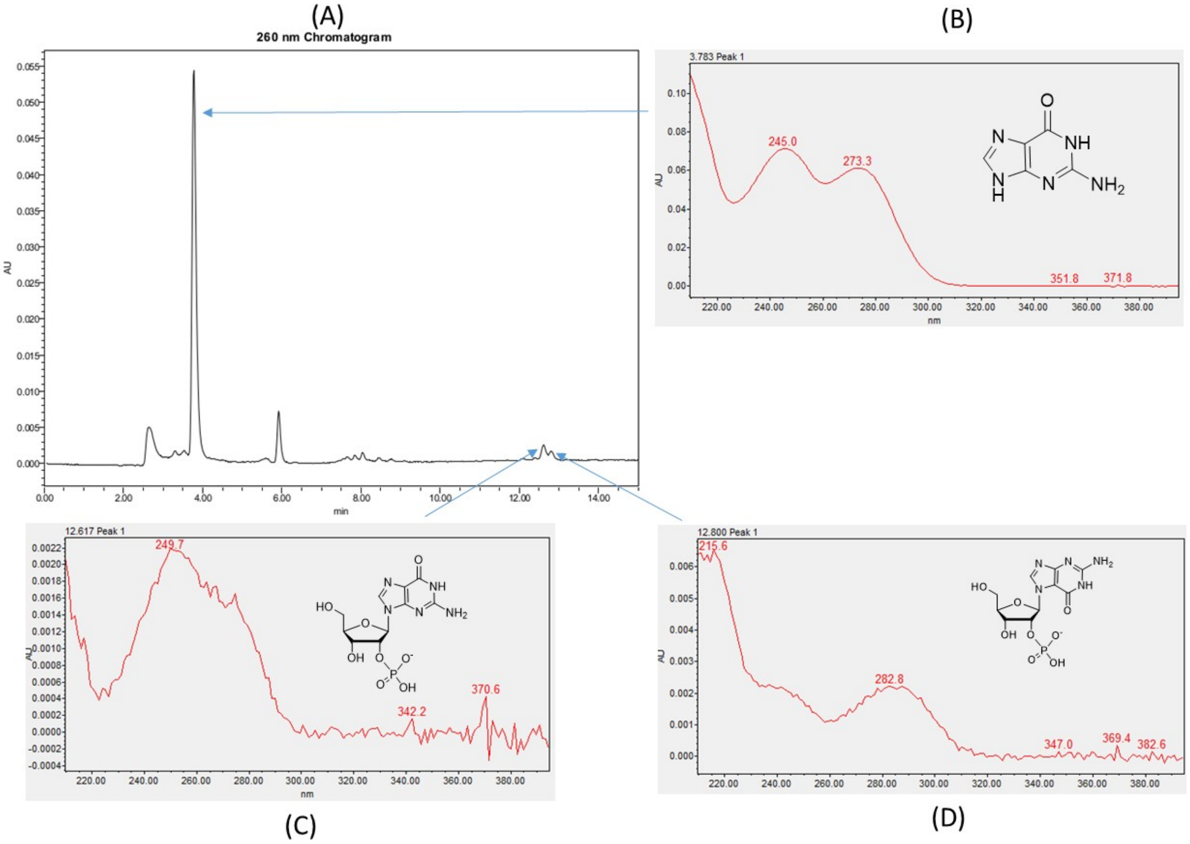

Fig. S9.

Fig. S6.

Formation of guanosine 2'-phosphate 7. (A) HPLC trace of the formation of guanosine 2'-phosphate **7** from the coupling of ribose 1,2-cyclic phosphate **3** and guanine**.** (B) Extracted UV spectrum of the peak at 3.8 min that showed absorption maximum at 245 nm. This UV is identical to guanine. (C) Extracted UV spectrum of the peak at 12.6 min that showed absorption maximum at 250 nm. This UV is identical to synthetic guanosine 2'-phosphate. (D) Extracted UV spectrum of the peak at 12.8 min that showed absorption maximum at 283 nm. This UV is identical to synthetic N^7-ribofuranosylguanine which indicated this compound is N^7-ribofuranosylguanine 2'-phosphate.

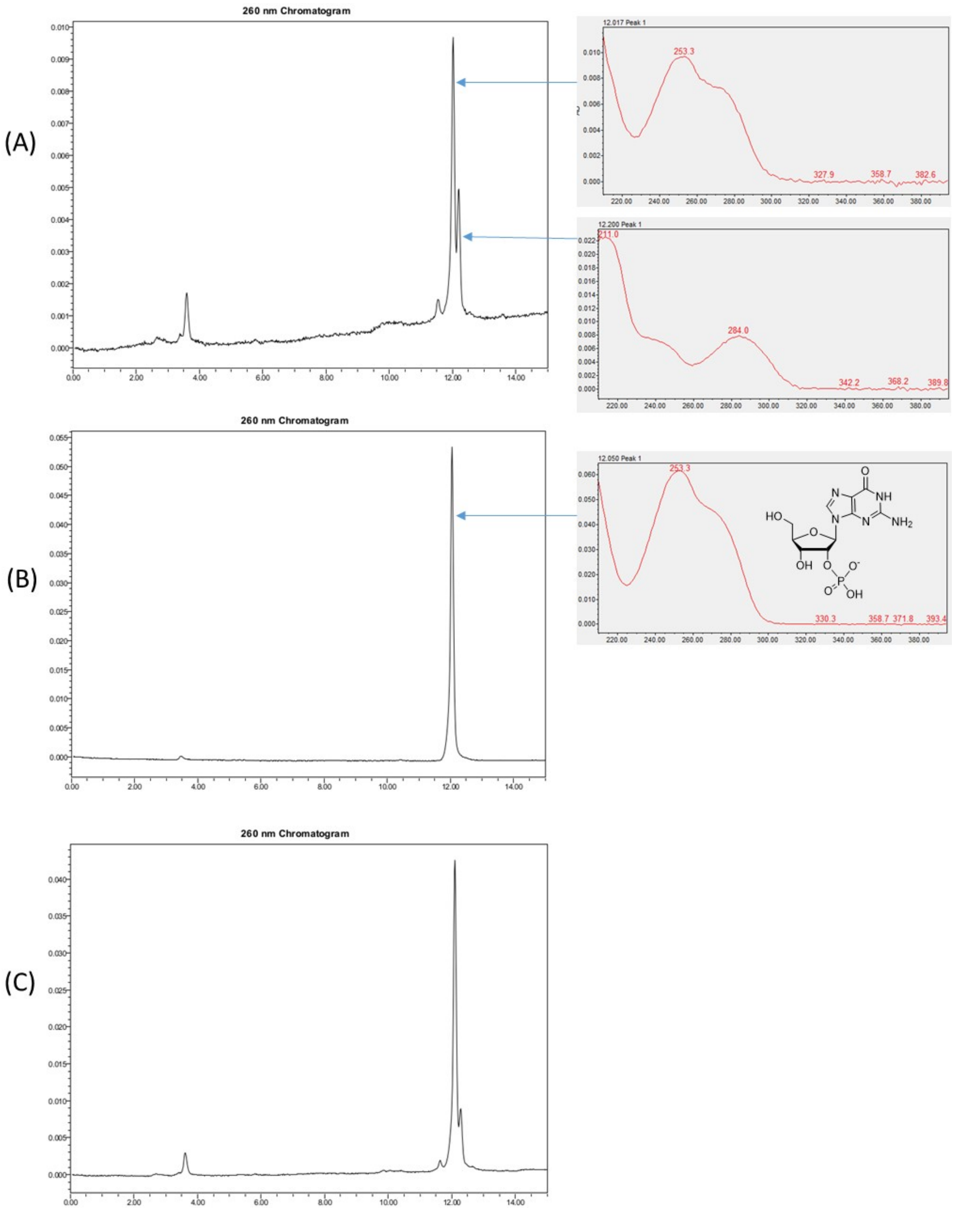

Fig. S10.

Formation of guanosine 2’-phosphate 7. (A) Ion exchange HPLC trace of the purified fraction (collection around 12 min) of the coupling of ribose 1,2-cyclic phosphate **3** and guanine and its UV spectrum**.** (B) Ion exchange HPLC of guanosine 2’-phosphate from chemical synthesis and its UV spectrum. (C) HPLC of co-injection of (A) and (B) which confirms the first peak of (A) and (B) are same compound.

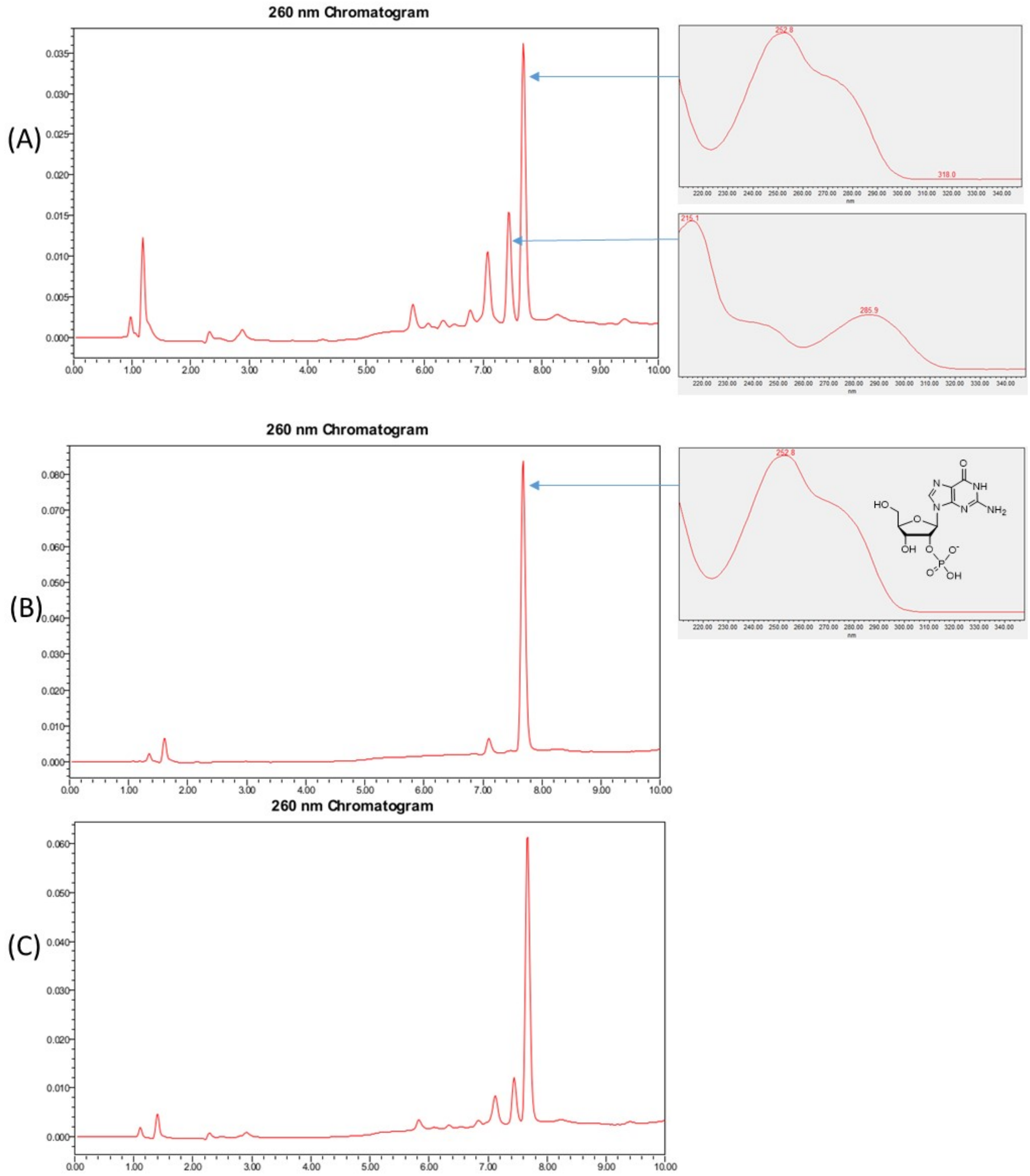

Fig. S11.

Formation of guanosine 2'-phosphate 7. (A) Reverse phase HPLC trace of the purified fraction (collection around 12 min of ion exchange HPLC) of the coupling of ribose 1,2-cyclic phosphate **3** and guanine and its UV spectrum**.** HPLC shows the major peak at 7.7 min. (B) Reverse phase HPLC trace of guanosine 2'-phosphate by chemical synthesis and its UV spectrum. (C) HPLC trace of co-injection of (A) and (B) which confirms the major peak of (A) and (B) are same compound.

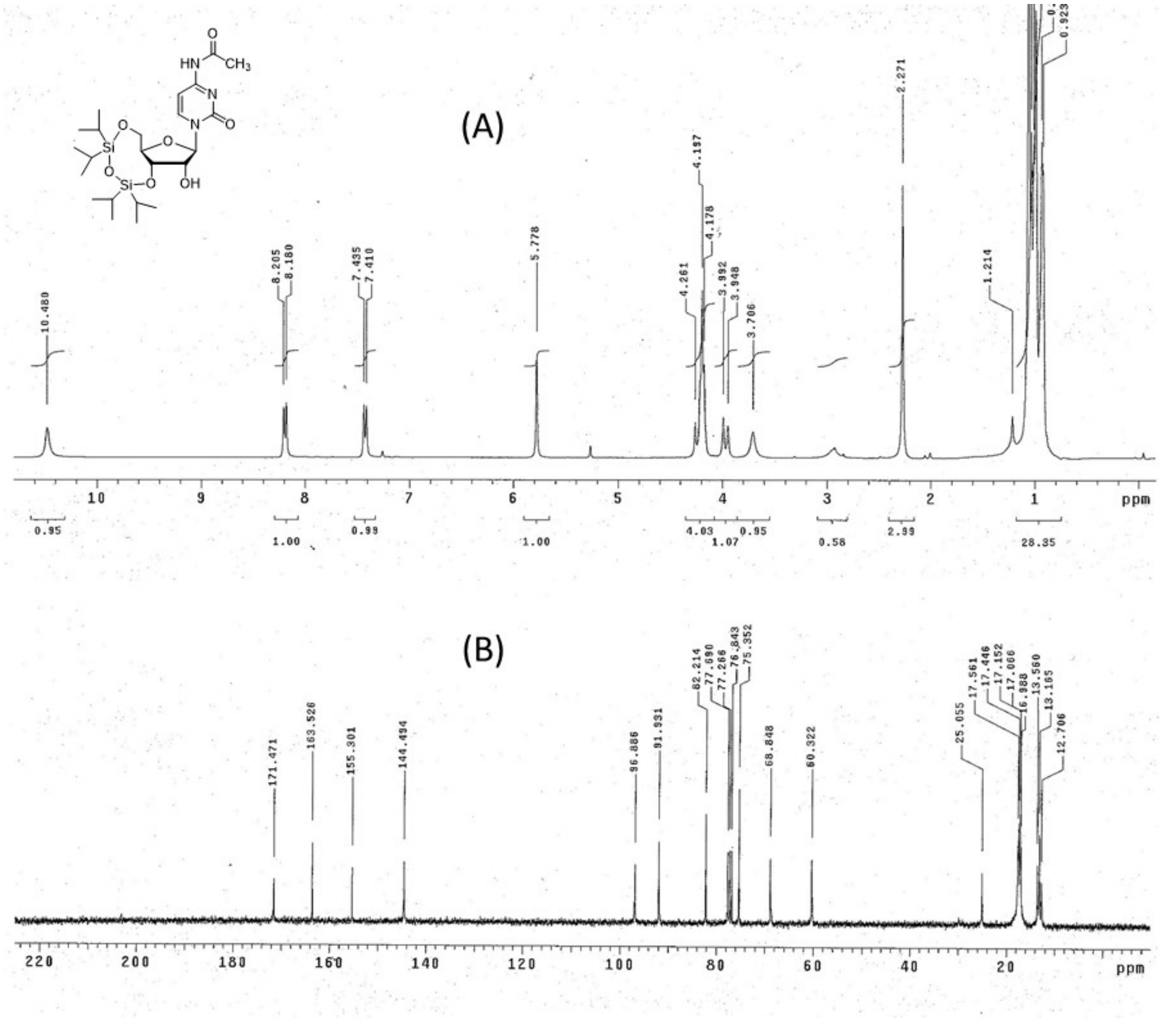

Fig. S12.

Spectral data of 10. (A) ^{1}H NMR spectrum of **10** (B) ^{13}C NMR spectrum of **10**

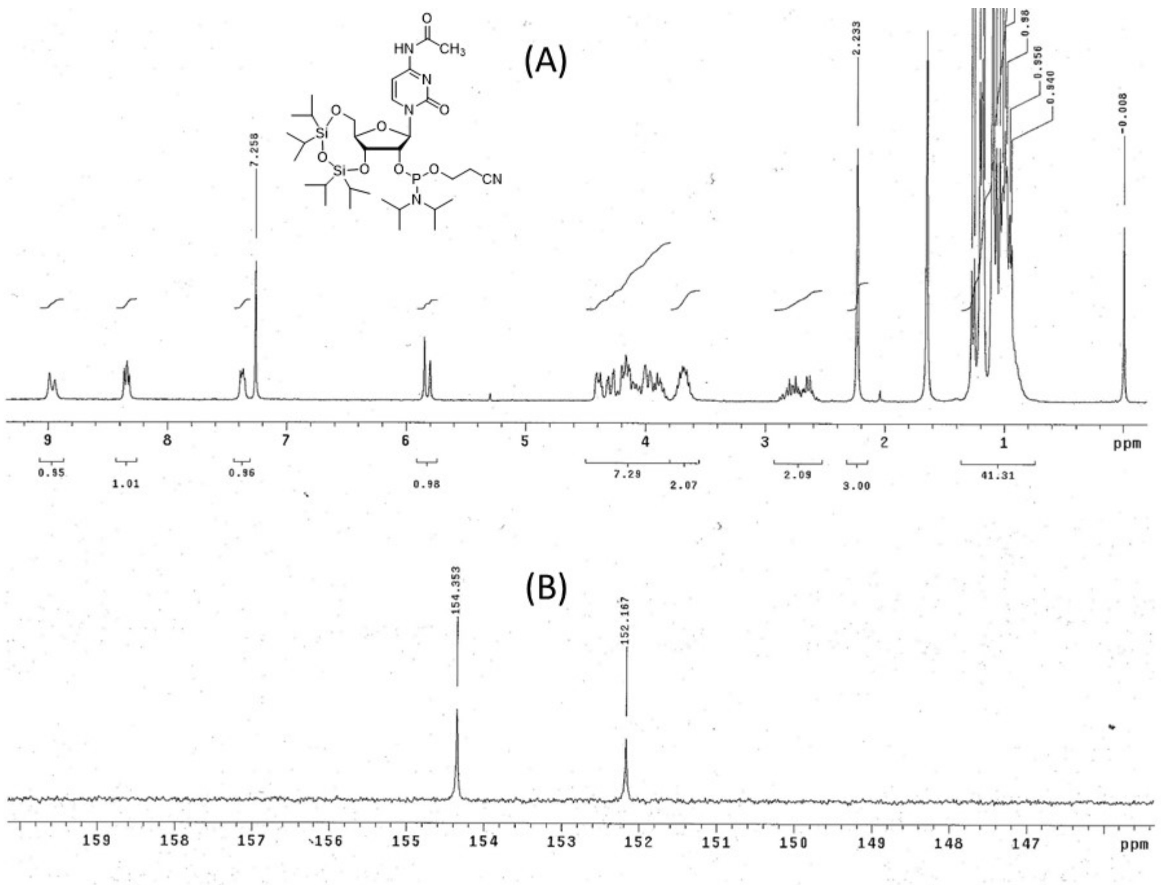

Fig. S13.
Spectral data of 12. (A) ^{1}H NMR spectrum of **12** (B) ^{31}P NMR spectrum of **12**

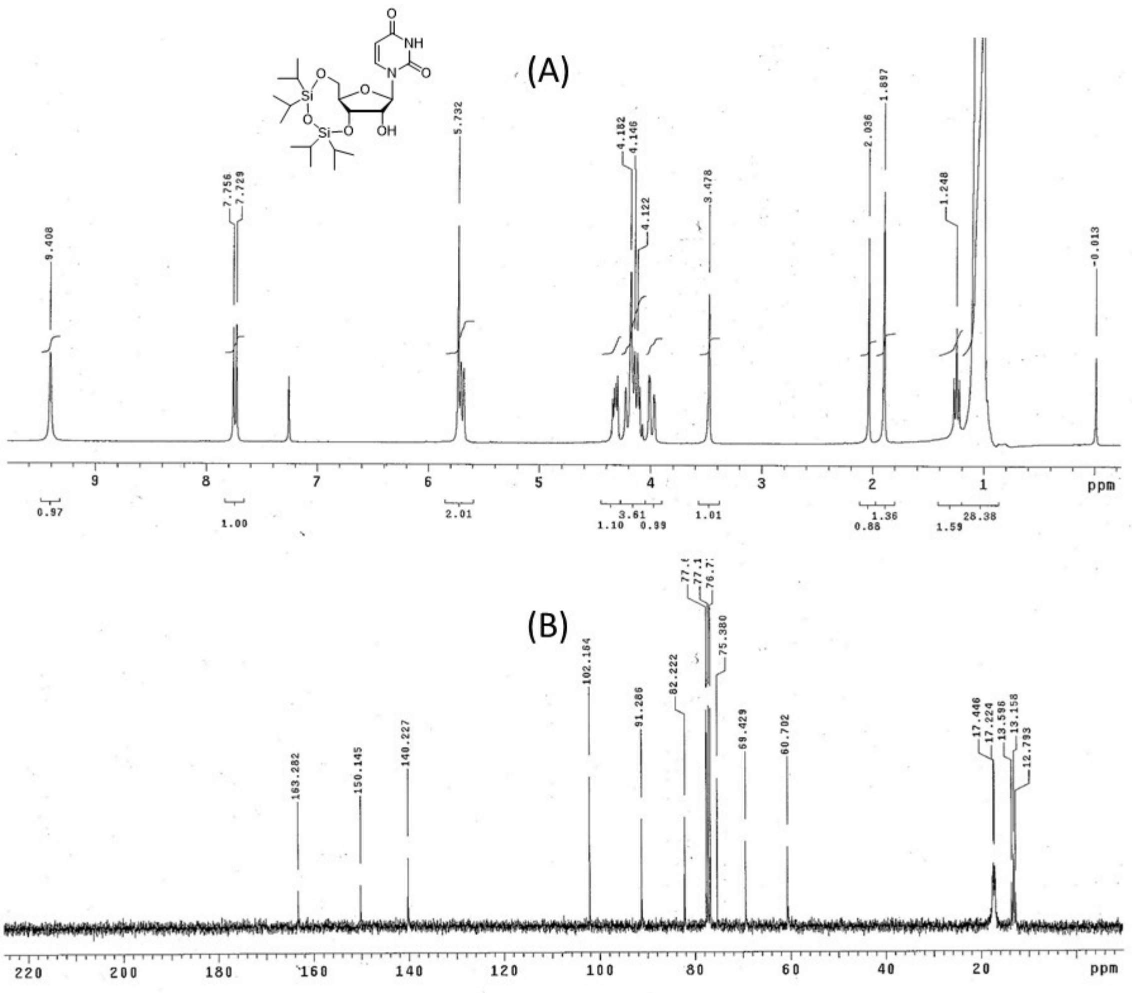

Fig. S14.

Spectral data of 16. (A) ^{1}H NMR spectrum of **16** (B) ^{13}C NMR spectrum of **16**

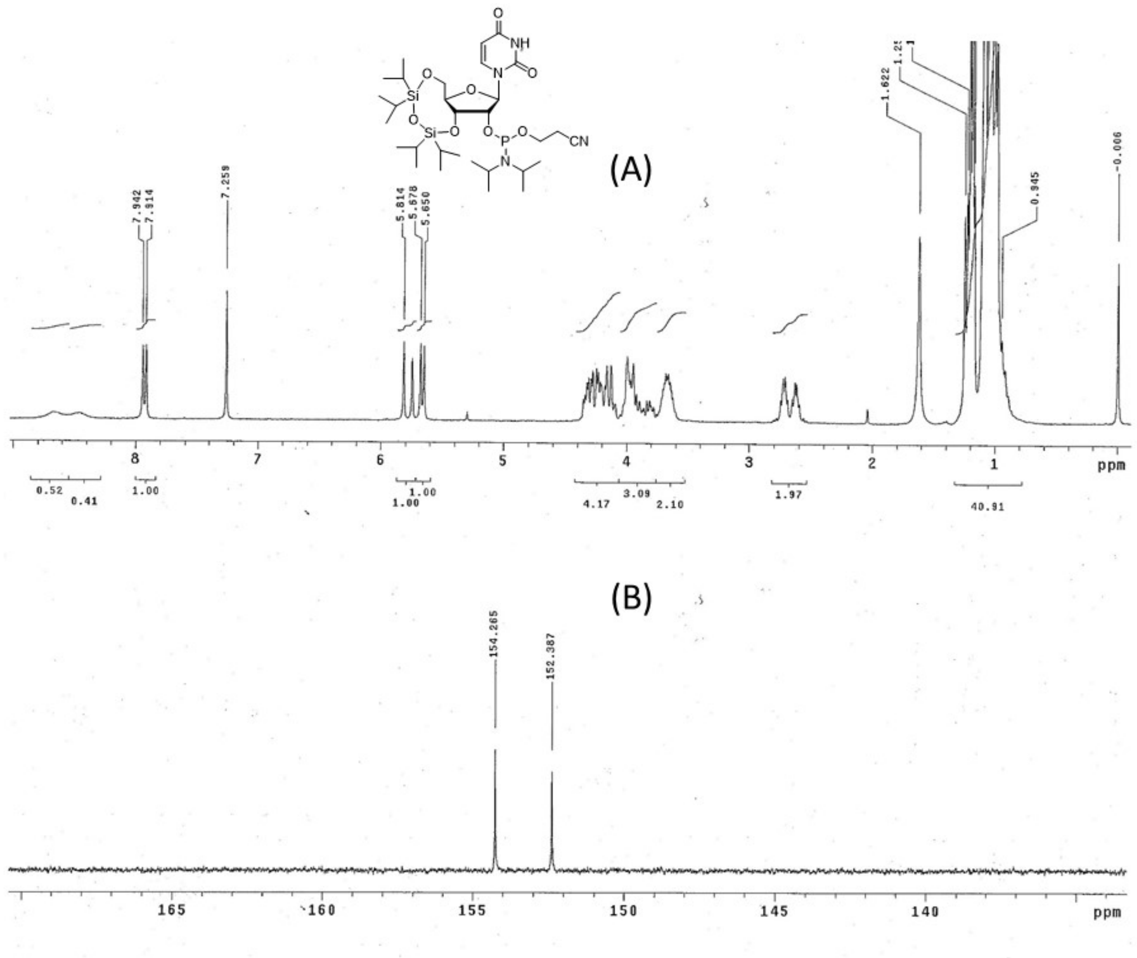

Fig. S15.
Spectral data of 17. (A) ^{1}H NMR spectrum of **17** (B) ^{31}P NMR spectrum of **17**

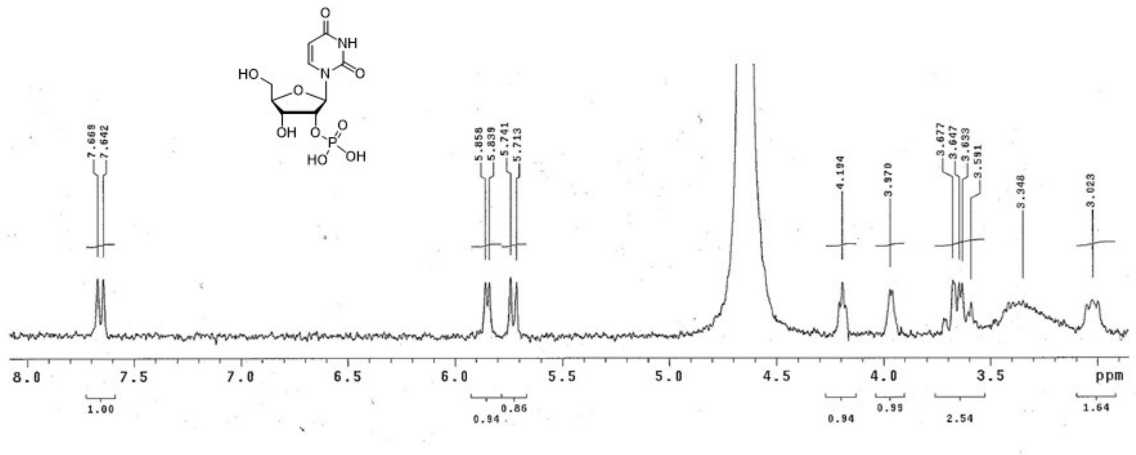

Fig. S16.
^{1}H NMR spectrum of 6 in D_2O

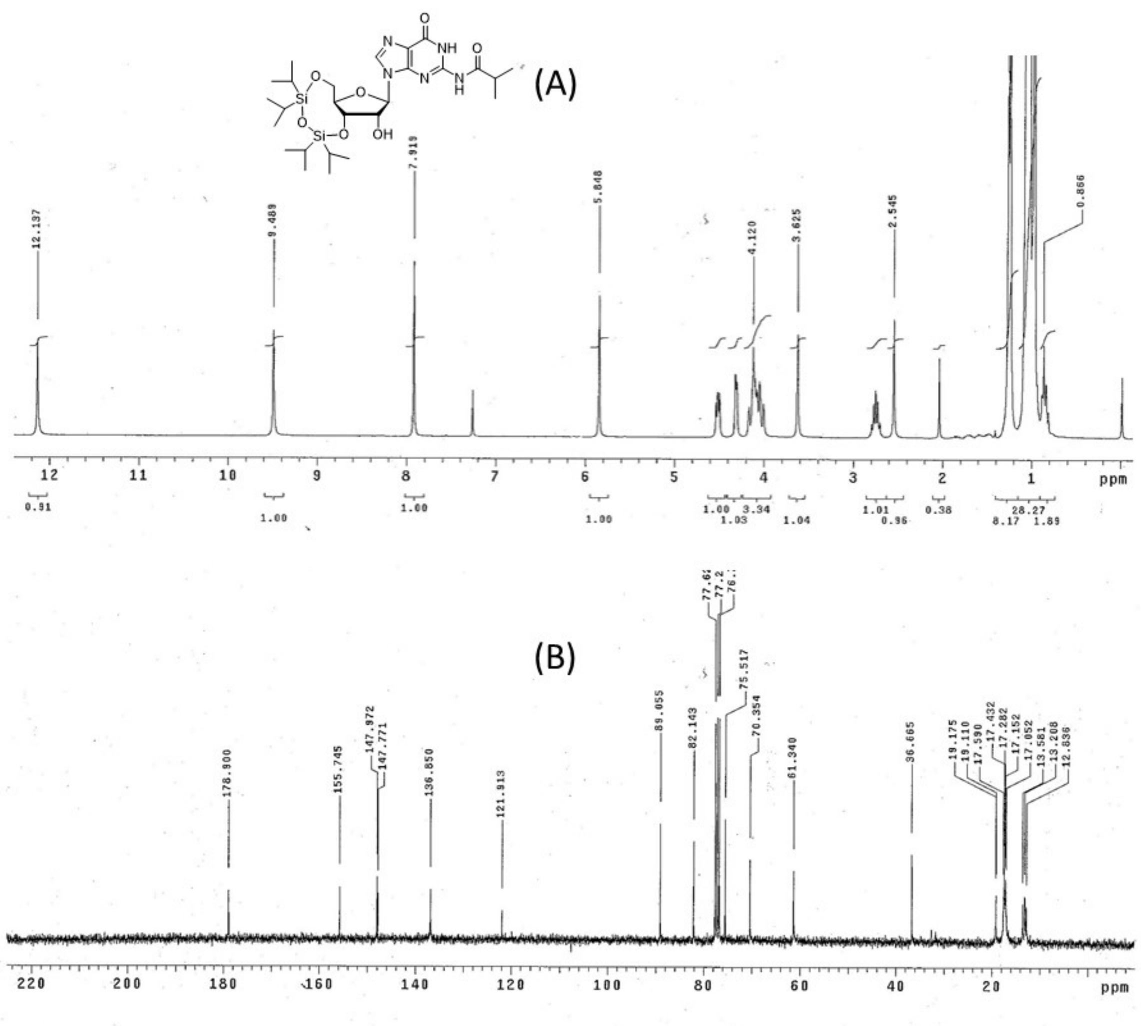

Fig. S17.

Spectral data of 19. (A) ^{1}H NMR spectrum of **19** (B) ^{13}C NMR spectrum of **19**

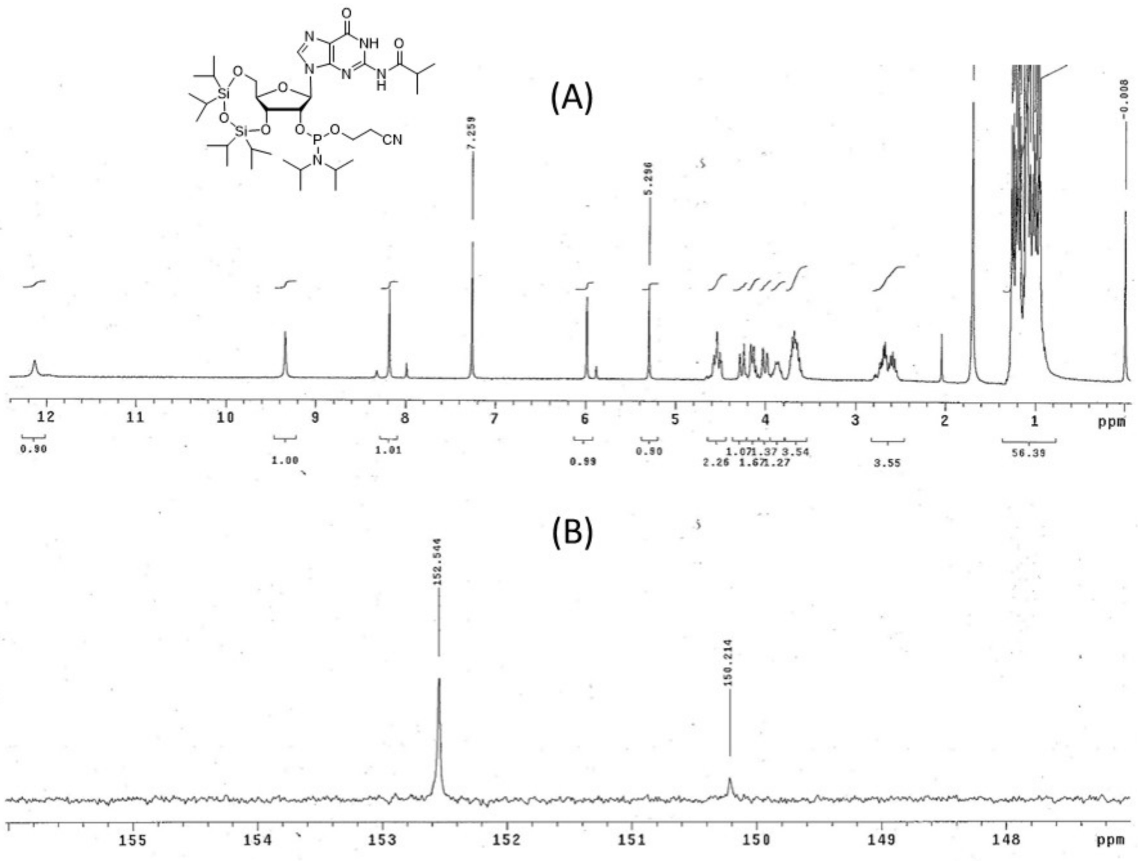

Fig. S18.

Spectral data of 20. (A) ^{1}H NMR spectrum of **20** (B) ^{31}P NMR spectrum of **20**

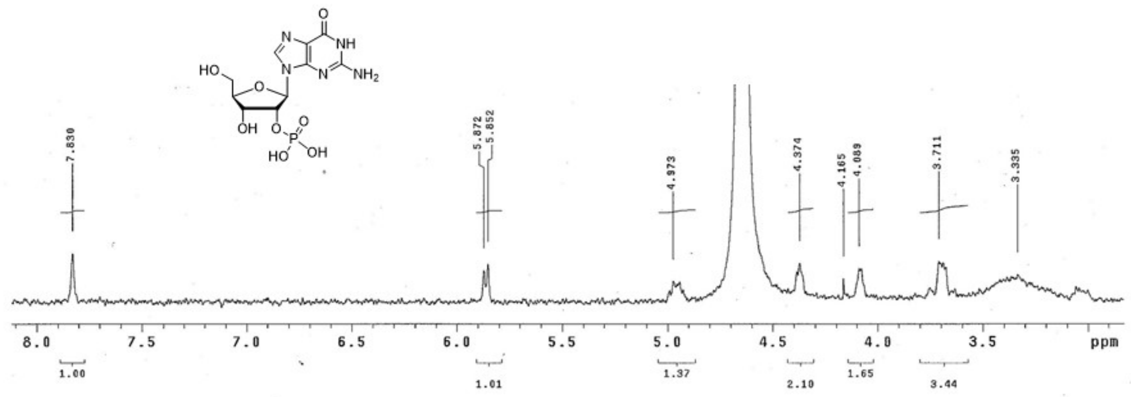

Fig. S19.
^{1}H NMR spectrum of 7 in D_2O

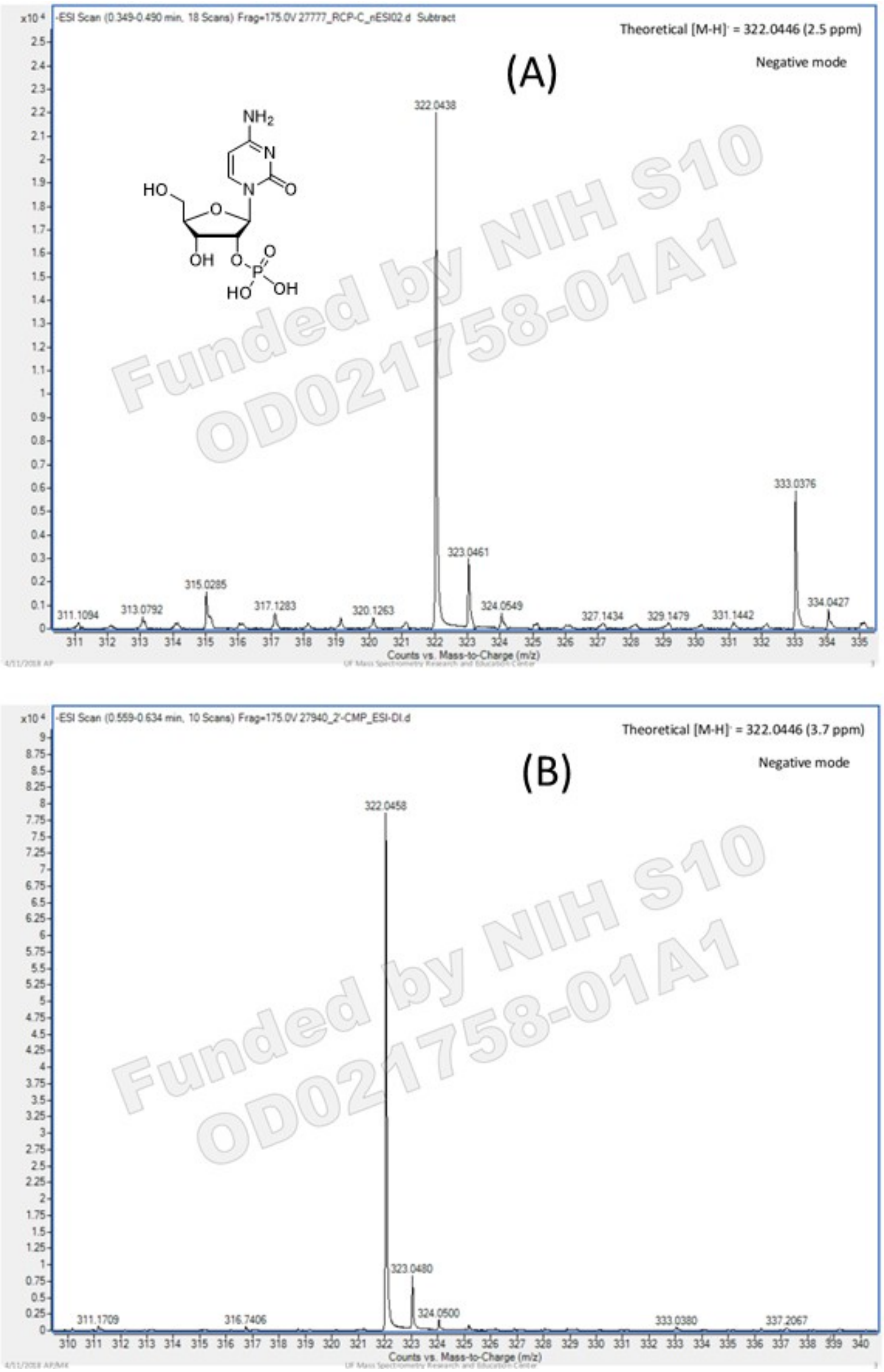

Fig. S20.

Mass spectrum data of 5. (A) Mass spectrum of **5** from the prebiotic reaction (B) Mass spectrum of **5** from chemical synthesis

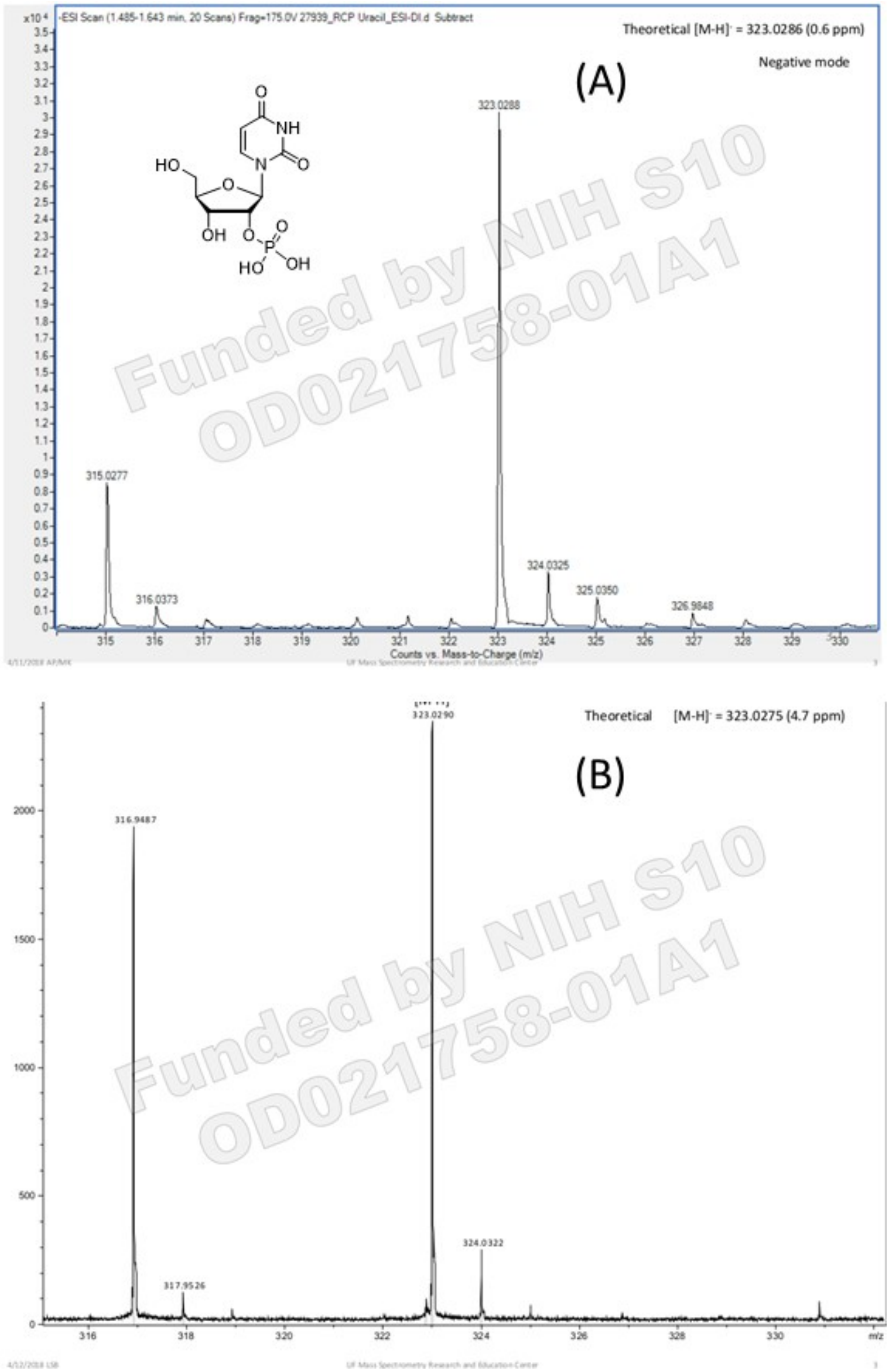

Fig. S21.

Mass spectrum data of 6. (A) Mass spectrum of **6** from the prebiotic reaction (B) Mass spectrum of **6** from chemical synthesis

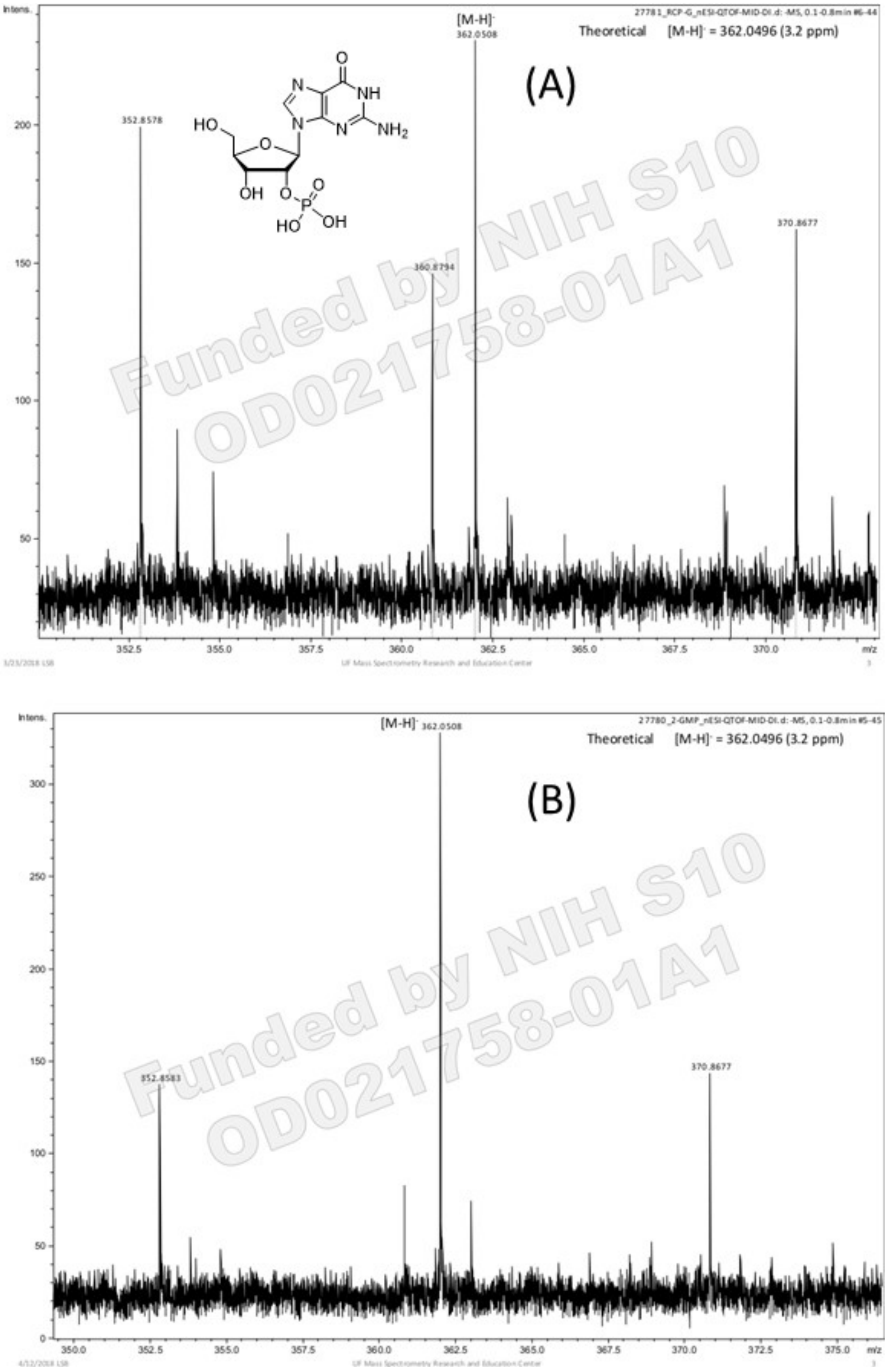

Fig. S22.

Mass spectrum data of 7. (A) Mass spectrum of **7** from the prebiotic reaction (B) Mass spectrum of **7** from chemical synthesis

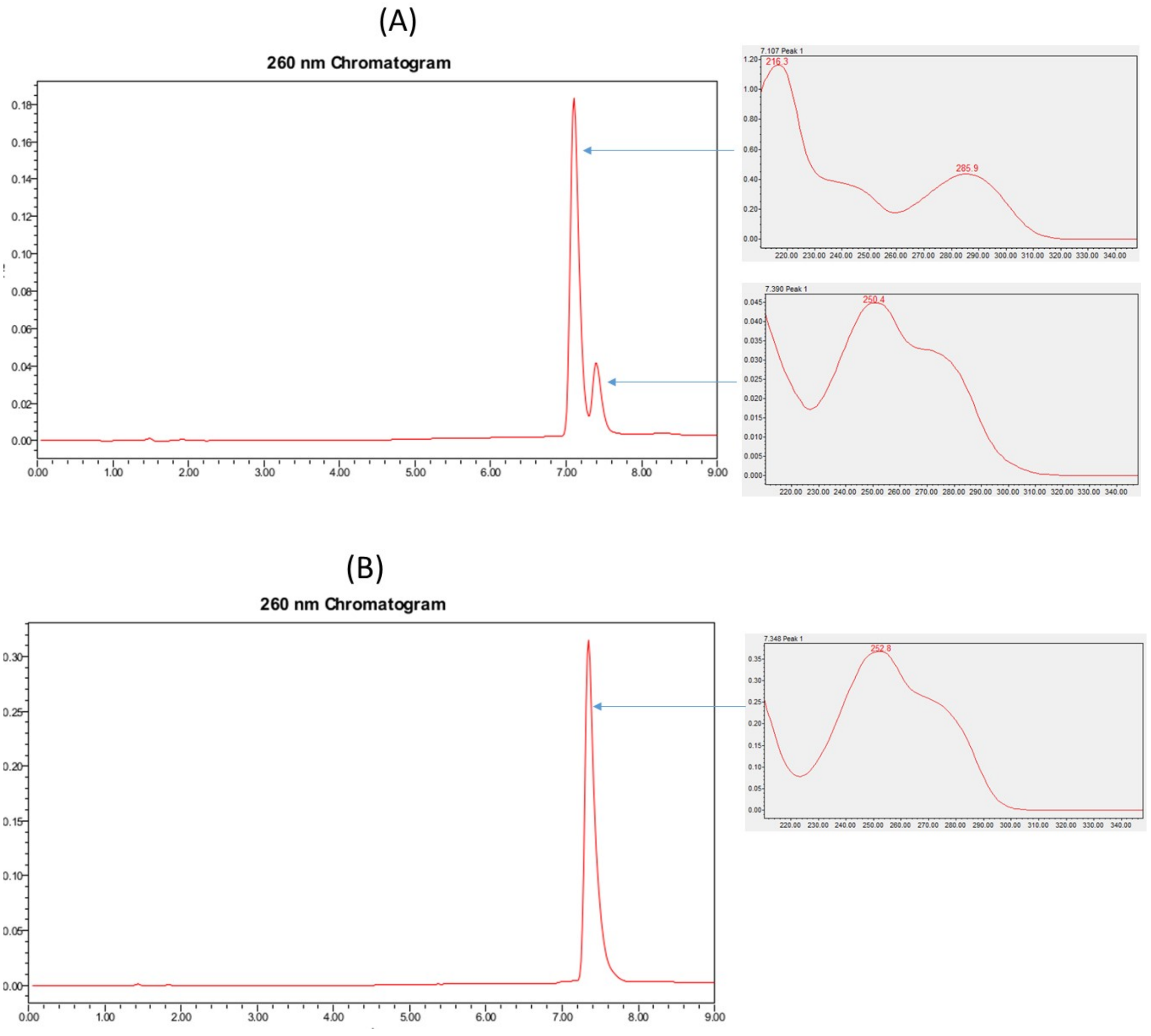

Fig. S23.

N^7-Ribofuranosylguanine. (A) HPLC trace of the mixture of N^7/N^9 ribofuranosylguanine from the chemical synthesis and its UV spectrum. The peak at 7.1 min is N^7-ribofuranosylguanine and the peak at 7.4 min is N^9-ribofuranosylguanine (guanosine). (B) HPLC trace of commercial guanosine (N^9-ribofuranosylguanine) and its UV spectrum.

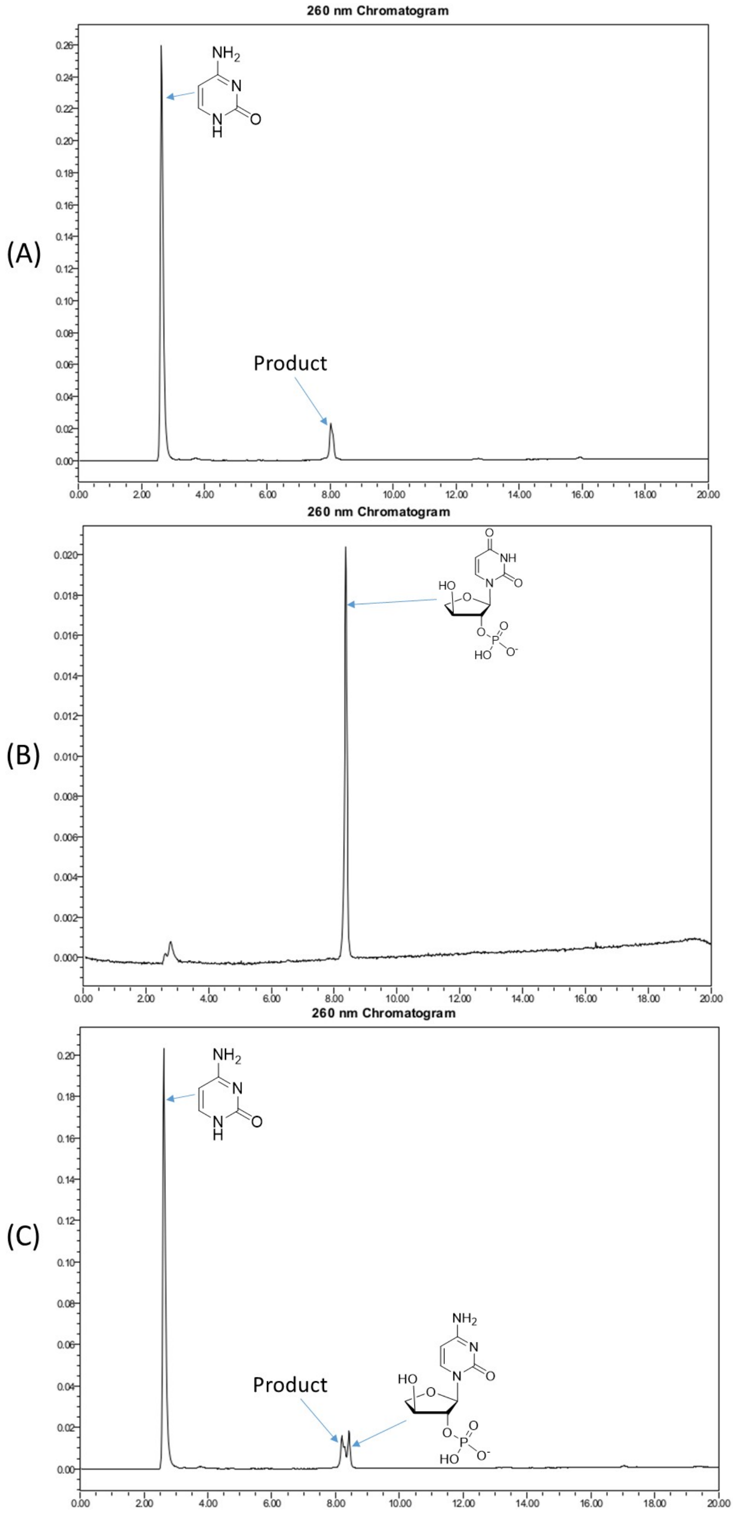

Fig. S24.

Attempted synthesis of threofuranosylcytosine 2’-phosphate 28. (A) HPLC trace of the coupling of threose 1,2-cyclic phosphate and cytosine with Ca^{2+} (B) HPLC trace of chemically synthesized threofuranosylcytosine 2’-phosphate (C) HPLC trace of co-

injection of (A) and (B) which shows the coupling product of threose 1,2-cyclic phosphate and cytosine and threofuranosylcytosine 2’-phosphate are different compounds.
